# Intrapulmonary (i.pulmon.) Pull Immunization With the Tuberculosis Subunit Vaccine Candidate H56/CAF01 After Intramuscular (i.m.) Priming Elicits a Distinct Innate Myeloid Response and Activation of Antigen-Presenting Cells Than i.m. or i.pulmon. Prime Immunization Alone

**DOI:** 10.3389/fimmu.2020.00803

**Published:** 2020-05-07

**Authors:** Aneesh Thakur, Fernanda Endringer Pinto, Harald Severin Hansen, Peter Andersen, Dennis Christensen, Christian Janfelt, Camilla Foged

**Affiliations:** ^1^Department of Pharmacy, Faculty of Health and Medical Sciences, University of Copenhagen, Copenhagen, Denmark; ^2^Department of Chemistry, Federal University of Espírito Santo, Vitoria, Brazil; ^3^Department of Drug Design and Pharmacology, Faculty of Health and Medical Sciences, University of Copenhagen, Copenhagen, Denmark; ^4^Department of Infectious Disease Immunology, Statens Serum Institut, Copenhagen, Denmark

**Keywords:** H56/CAF01, tuberculosis, subunit vaccine, pulmonary administration, myeloid cells, antigen-presenting cells, mass spectrometry imaging, drug delivery

## Abstract

Understanding the *in vivo* fate of vaccine antigens and adjuvants and their safety is crucial for the rational design of mucosal subunit vaccines. Prime and pull vaccination using the T helper 17-inducing adjuvant CAF01 administered parenterally and mucosally, respectively, has previously been suggested as a promising strategy to redirect immunity to mucosal tissues. Recently, we reported a promising tuberculosis (TB) vaccination strategy comprising of parenteral priming followed by intrapulmonary (i.pulmon.) mucosal pull immunization with the TB subunit vaccine candidate H56/CAF01, which resulted in the induction of lung-localized, H56-specific T cells and systemic as well as lung mucosal IgA responses. Here, we investigate the uptake of H56/CAF01 by mucosal and systemic innate myeloid cells, antigen-presenting cells (APCs), lung epithelial cells and endothelial cells in mice after parenteral prime combined with i.pulmon. pull immunization, and after parenteral or i.pulmon. prime immunization alone. We find that i.pulmon. pull immunization of mice with H56/CAF01, which are parenterally primed with H56/CAF01, substantially enhances vaccine uptake and presentation by pulmonary and splenic APCs, pulmonary endothelial cells and type I epithelial cells and induces stronger activation of dendritic cells in the lung-draining lymph nodes, compared with parenteral immunization alone, which suggests activation of both innate and memory responses. Using mass spectrometry imaging of lipid biomarkers, we further show that (i) airway mucosal immunization with H56/CAF01 neither induces apparent local tissue damage nor inflammation in the lungs, and (ii) the presence of CAF01 is accompanied by evidence of an altered phagocytic activity in alveolar macrophages, evident from co-localization of CAF01 with the biomarker bis(monoacylglycero)phosphate, which is expressed in the late endosomes and lysosomes of phagocytosing macrophages. Hence, our data demonstrate that innate myeloid responses differ after one and two immunizations, respectively, and the priming route and boosting route individually affect this outcome. These findings may have important implications for the design of mucosal vaccines intended for safe administration in the airways.

## Introduction

Tuberculosis (TB) is one of the top 10 causes of death worldwide, and the disease killed an estimated 1.3 million people in 2017 (1). Approximately one fourth of the world’s population is latently infected with *Mycobacterium tuberculosis* (*Mtb*), and these individuals remain susceptible to active TB for the rest of their life (1). With the emergence of multi-drug resistant *Mtb* strains, a novel vaccine, which is more effective than the currently available Bacillus Calmette-Guérin (BCG) vaccine, is required to achieve the World Health Organization’s important goal of ending the global TB epidemic by 2035 (2). In this respect, mucosal delivery via intrapulmonary (i.pulmon.) administration of subunit vaccines having excellent safety profiles (3, 4) is a promising strategy to induce protective lung-localized *Mtb*-specific T-cell responses (5). However, little is known about the *in vivo* fate of inhaled vaccine antigens and adjuvants, and their safety.

Innate myeloid cells include mononuclear phagocytes, monocytes, dendritic cells (DCs), and granulocytes. These cells play essential roles in pathogen clearance, initiation, regulation and resolution of inflammation, and antigen presentation (6, 7). Following repeated immunizations, i.e., prime – pull immunization strategies, there is a continuous cross-talk between innate and adaptive immune cells and vaccine components. Hence, knowledge about these events is crucial to improve the immunogenicity, protective efficacy and safety of vaccines. Recent advances in the understanding of the diversity of myeloid and non-myeloid antigen-presenting cells (APCs) clearly suggest that for vaccines to induce specific immune profiles, they should be targeted to immune cell subsets capable of inducing that specific type of immune response (8, 9). For different subunit vaccines administered i.pulmon., inconsistencies exist in the immune responses they induce, and these differences may be due to factors like (i) the diversified localization of different APC subsets in the respiratory tract and the lung-draining lymph nodes (LNs), (ii) their functional differences, (iii) the size of the antigen, and (iv) the physicochemical properties of the adjuvant (10–13). Therefore, an understanding of the initial interactions taking place between the vaccine [antigen(s) and adjuvant] and the immune system is crucial for the rational design of safe vaccines, which have the capability to induce long-lasting protective immunity in the lungs (14).

The subunit vaccine antigen H56 is a fusion protein composed of the *Mtb* antigens Ag85B, ESAT-6, and Rv2660c, and in combination with the cationic adjuvant formulation 01 (CAF01) administered parenterally, this antigen elicits a polyfunctional Th1/Th17 CD4^+^ T cell response and causes a significant reduction in *Mtb* burden (15–17). CAF01 is composed of cationic liposomes based on the surfactant dimethyldioctadecylammonium (DDA) bromide and the glycolipid trehalose-6,6’-dibehenate (TDB) (18). CAF01 delivers antigen and activates DCs (19), induces both humoral and cell-mediated memory immune responses, and it has been tested in phase I clinical trials, demonstrating an excellent safety and immunogenicity profile (20–22). Our recent data suggests that a parenteral prime – mucosal pull immunization strategy using CAF01 can be applied to redirect immunity to mucosal tissues (23). Recently, we reported an immunization strategy comprising intramuscular (i.m.) priming followed by i.pulmon. mucosal pull immunization with the H56/CAF01 vaccine, which resulted in the induction of lung-localized, H56-specific T cells and systemic as well as lung mucosal IgA responses (24). However, the role of (i) H56/CAF01 deposition within lung tissue, (ii) CAF01 internalization by phagocytic cells, and (iii) antigen presentation in the lungs and the lung-draining LNs on the induction of immune responses after pulmonary administration are unknown. In addition, an outstanding research question is the safety of CAF01 upon pulmonary administration.

Here, we applied multicolor flow cytometry to investigate H56/CAF01 uptake by innate myeloid cells and APCs in the lungs, the spleen, and the lung-draining LNs in mice after i.m. or i.pulmon. prime or i.m. prime – i.pulmon. pull immunization. We compared homologous prime – pull immunization with prime immunization alone to examine if pre-existing systemic H56-specific immunity induced by H56/CAF01 leads to different safety issues as compared to pulmonary prime immunization alone. We did not include mucosal prime – boost immunization as previous studies have showed no overt immunological advantage applying this immunization strategy (25). Mass spectrometry imaging (MSI) was used to follow the time-dependent biodistribution of CAF01 and selected lipid biomarkers in lung tissue.

## Materials and Methods

### Materials

Dimethyldioctadecylammonium was obtained from Avanti Polar Lipids (Alabaster, AL, United States). TDB was purchased from Niels Clauson-Kaas A/S (Farum, Denmark). Xenolight 1,1′-dioctadecyl-3,3,3′,3′-tetramethylindotricarbocyanine iodide (DiR) near infra-red fluorescent dye was purchased from Perkin Elmer (Waltham, MA, United States). H56 protein was produced recombinantly in *E. coli* as previously described (15), reconstituted in 20 mM glycine buffer (pH 8.8), checked for purity, and validated for residual DNA, endotoxins and bioburden following internal good manufacturing practice standards at Statens Serum Institut as described previously (16). Alexa Fluor^®^ 647-labeling of H56 was performed commercially (Thermo Fischer Scientific, Eugene, OR, United States). 2,5-dihydroxybenzoic (DHB), 1,5-diaminonaphthalene (DAN), trifluoroacetic acid (TFA), and Meyer’s hematoxylin solution and eosin (H&E) solution were purchased from Sigma-Aldrich (St. Louis, MO, United States). Methanol was obtained from Th. Geyer (Renningen, Germany). Water was prepared by using a Millipore Direct-Q3 UV system (Billerica, MA, United States). All other chemicals and reagents were of analytical grade and were acquired from commercial suppliers.

### Preparation of CAF01

Liposomes were prepared by using the thin film method and characterized for average intensity-weighted hydrodynamic diameter (*z*-average), polydispersity index (PDI) and zeta-potential as previously described (24). Briefly, weighed amounts of DDA and TDB (5:1, w/w) were dissolved in chloroform/methanol (9:1, v/v) in a round bottom flask. The lipid mixture was dried overnight by rotary evaporation under vacuum after cleaning with 99% (v/v) ethanol. The lipid film was rehydrated in 10 mM Tris buffer (pH 7.4), sonicated for 5 min using an ultrasound cleaner (Branson Ultrasonic Cleaner, Danburry, CT, United States), and heated to 60°C for 1 h in a water bath with vortexing every 10 min. The liposomes were tip-sonicated for 20 s, 20 min after the rehydration by using a MISONIX S-4000 probe sonicator (LLC, Newtown, CT, United States) (amplitude 70; power 16 W) to reduce their size. The final concentration of CAF01 was 20/4 mg/mL of DDA/TDB, corresponding to a molar ratio of 89:11. Solutions of unlabeled or Alexa Fluor^®^ 647-labeled H56 were mixed with equal volumes of CAF01 dispersions at concentrations of 5 and 10 μg/mL, respectively, and the antigen was allowed to adsorb to the liposomes for 30 min at room temperature before administration. Fluorescently labeled CAF01 was prepared by addition of Xenolight DiR dissolved in ethanol during the preparation of the lipid film, resulting in a DiR concentration of 0.025 mg/mL in the final formulation.

### Immunizations

Six-to-eight-week old female BALB/c mice (Scanbur, Karlslunde, Denmark) were allowed to acclimatize for 1 week upon arrival. All experimental work was approved by the Danish National Experiment Inspectorate under permit 2016-15-0201-01026 and was performed in accordance with the European Community directive 86/609 for the care and use of laboratory animals. Mice (6–12/group) were immunized once by i.m. or i.pulmon administration, or by i.m. priming followed by i.pulmon. pull immunization after an interval of 2 weeks. For the i.m. immunizations, 5 μg Alexa Fluor^®^ 647-labeled unadjuvanted H56 or 5 μg Alexa Fluor^®^ 647-labeled H56 adjuvanted with DiR-labeled CAF01 (250/50 μg DDA/TDB) was injected in the right thigh muscles. For the i.pulmon. immunizations, 10 μg Alexa Fluor^®^ 647-labeled unadjuvanted H56 or 10 μg Alexa Fluor^®^ 647-labeled H56 adjuvanted with DiR-CAF01 (125/25 μg DDA/TDB) was used, and they were performed as described previously (24). All vaccines were formulated and administered in a dose-volume of 50 μL in isotonic Tris buffer. Mice dosed i.m. or i.pulmon. with 50 μL isotonic Tris buffer served as negative controls.

### Organ Collection and Cell Preparation

Mice were euthanized 3, 24, or 72 h after the immunizations, and the lungs, spleens, and draining LNs (inguinal and popliteal LNs to which vaccines administered i.m. are draining, and the tracheobronchial and mediastinal LNs to which vaccines administered i.pulmon. are draining) were isolated. For the MSI study, mice were euthanized after 6, 24, 48, and 72 h, and 7, 10, and 14 days. To collect lung tissue, a previously described protocol was used that involves flushing the pulmonary circulation and inflating the lung with dispase during tissue harvest, followed by homogenization and digestion in a DNase and collagenase solution (26). Briefly, following euthanasia of mice, the trachea was intubated transorally. The pulmonary circulation was flushed with 2 mL phosphate-buffered saline (PBS) followed by instillation of 1 mL dispase (Corning Life Sciences, Tewksbury, MA, United States). Subsequently, a volume of 0.7 mL dispase was administered *via* the endotracheal catheter, followed by administration of 0.5 mL 1% (w/v) low-melting-point agarose (Sigma-Aldrich, Brøndby, Denmark) heated to 50°C, which served as a semi-solid plug when cooled to keep the enzyme solution in close proximity to the lung tissue. The lungs were excised, placed in RPMI 1640 (Sigma-Aldrich) at 4°C, and dissociated in gentleMACS C tubes (Miltenyi Biotec Norden, Lund, Sweden) containing 2 mL RPMI 1640, 5% (v/v) fetal calf serum (FCS, Gibco Thermo Fisher, Hvidovre, Denmark), 1.5 mg/mL collagenase type IV (Sigma-Aldrich), and 20 units/mL DNase (Sigma-Aldrich) by using the gentleMACS dissociator (Miltenyi Biotec Norden AB). After 1 h incubation at 37°C, the lung pieces were dissociated again by using the gentleMACS dissociator and centrifuged at 700 × *g* for 5 min. Lung cell pellets were forced through a 70 μm cell-strainer (Falcon, Durham, NC, United States) and washed twice with RPMI 1640. Single cell suspensions of splenocytes were obtained by homogenizing the spleens through a nylon-mesh cell-strainer (Falcon) followed by two washings with RPMI 1640. The LNs were treated with 2 mL RPMI supplemented with 1 mg/mL Collagenase type IV and 20 units/mL DNase. After 30 min of incubation at 37°C, the LNs were passed through the nylon-mesh cell-strainer, followed by two washings with RPMI 1640. For each lung, spleen or LN, 1 × 10^6^ cells (or everything, if the sample contained less cells) were resuspended in RPMI 1640 supplemented with 5 × 10^–5^ M 2-mercaptoethanol (Gibco Thermo Fisher), 1% (v/v) sodium pyruvate (Sigma-Aldrich), 1% (v/v) penicillin-streptomycin (Gibco Thermo Fisher), 1% (v/v) HEPES (Gibco Thermo Fisher), and 10% (v/v) FCS (Gibco Thermo Fisher) and transferred to 96-well, V-bottomed plates.

### Flow Cytometry

Single cell suspensions of lungs, spleen, and LNs from immunized mice were washed with PBS and resuspended in FACS-buffer [PBS supplemented with 1% (v/v) fetal calf serum and 0.1% (w/v) sodium azide]. Following treatment with Fc-block (BD Biosciences, Lyngby, Denmark), the cells were stained for 30 min at 4°C for surface markers using mAbs (Supplementary Table S1). Dead cells were excluded by using the fixable viability dyes FVS510 or FVS700 (BD). The cells were washed twice, resuspended in FACS buffer and analyzed using an LSRFortessa flow cytometer (BD). Gates for the surface markers are based on fluorescence-minus-one controls. The gating strategy used for identifying distinct cell populations in the lungs, the spleen, and the draining lymph nodes is based on previous reports (26–28) and is further described in the Supplementary Figures S1–S3. All flow cytometry analyses were performed using the FlowJo software v10 (Tree Star, Ashland, OR, United States).

### Tissue Preparation for Matrix-Assisted Laser Desorption/Ionization Mass Spectrometry Imaging (MALDI-MSI)

Following euthanasia, the lungs were removed, snap-frozen on crushed dry ice and stored at −80°C until analysis. Analysis time points selected for imaging were 6, 24, 48, 72, and 96 h and 7, 10, and 14 days with one mouse analyzed at each time point and one control animal. The frozen lungs were mounted onto a cryo-microtome sample specimen disk (Leica Biosystems Inc., Buffalo Grove, IL, United States) with 5% (w/v) carboxymethyl cellulose aqueous gel (Sigma-Aldrich) at −24°C. The lungs were cut into coronal sections of 18 μm thickness using a Leica CM3050S cryo-microtome (Leica Microsystems, Wetzlar, Germany), thaw-mounted onto microscope glass slides (VWR, Søborg, Denmark) and stored in a −80°C freezer until MSI analysis.

Prior to matrix application, the tissue sections were transferred directly from the freezer to a vacuum desiccator for 10 min. For the positive ion mode analysis, a solution of freshly prepared 30 mg/mL DHB in methanol/water (50:50, v/v) supplemented with 1% (w/v) TFA was used. For the negative ion mode analysis, a freshly prepared solution of 3 mg/mL DAN in methanol/water (90:10, v/v) was used. A volume of 300 μL matrix solution was sprayed onto the surface of the tissue sections using an in-house built pneumatic sprayer with the sample rotating at 150 rpm (for the application of the DHB matrix) or 250 rpm (for the application of the DAN matrix), and the matrix solution was pneumatically sprayed at a flow rate of 30 μL/min using a nebulizer gas pressure of 2 bar (29). The quality of matrix deposition (homogeneity and crystal size) was checked by inspection with reflected light optical microscopy.

### MALDI-MSI and Image Analysis

The samples were analyzed using a Thermo Q Exactive Orbitrap mass spectrometer (Thermo Scientific, Bremen, Germany) equipped with an AP-SMALDI10 ion source (TransMIT, Giessen, Germany). The AP-SMALDI10 ion source was equipped with a nitrogen laser with a wavelength of 337 nm, and a frequency of 60 Hz, operated using 30 laser pulses per pixel. Analysis was performed in the positive and negative ion modes, respectively, using a scan range of 300–1200 Da, a mass resolving power of 140,000 at *m/z* 200, a lock mass of *m/z* 431.037 corresponding to a signal from the DHB matrix in the positive ion mode, and a lock mass of *m/z* 311.130 corresponding to a signal from DAN matrix in the negative ion mode. Images were acquired at a pixel size of 100 μm with the ablation craters well separated. After imaging, the sections were stained with H&E as described in detail elsewhere (30), and images were acquired using an optical microscope. The raw files were converted to imzML files (31), and the MSiReader program was used for image generation (32). Images were generated with a bin width of ±0.002 Da (±5 ppm). Semi-quantitative data analysis (i.e., intensity ratio) was performed for measuring changes in the abundance of CAF01 lipids [DDA (m/z 550.629) and TDB (m/z 1025.726)] relative to endogenous tissue lipids, i.e., phosphatidylcholine {[PC (34:1)], m/z 798.541} and phosphatidylserine {[PS (38:4)], m/z 810.529}. For calculating the intensity ratio, a region of interest (ROI) was manually selected along the lung section, and the mean intensity of the lipid (i.e., DDA or TDB) in the ROI was divided by the mean intensity of endogenous tissue lipid (PC or PS) in that specific ROI. We also analyzed the distribution of phospholipids, i.e., lysophosphatidylcholines (LysoPC) and bis(monoacylglycero)phosphate (BMP), sphingolipids (ceramides), and cholesteryl esters in the lungs following i.pulmon. administration of CAF01.

### Statistical Analysis

Two-way ANOVA followed by Tukey’s multiple comparisons test was used to analyze the difference between the immunization groups using the GraphPad Prism software (GraphPad Software Inc., La Jolla, CA, United States). A value of *p* < 0.05 was considered significant.

## Results

### Mucosal (i.pulmon.) Pull Immunization of Mice Parenterally (i.m.) Primed With H56/CAF01 Induces Higher Vaccine Uptake by Pulmonary APCs as Compared to i.m. or i.pulmon. Priming Alone

**FIGURE 1:**
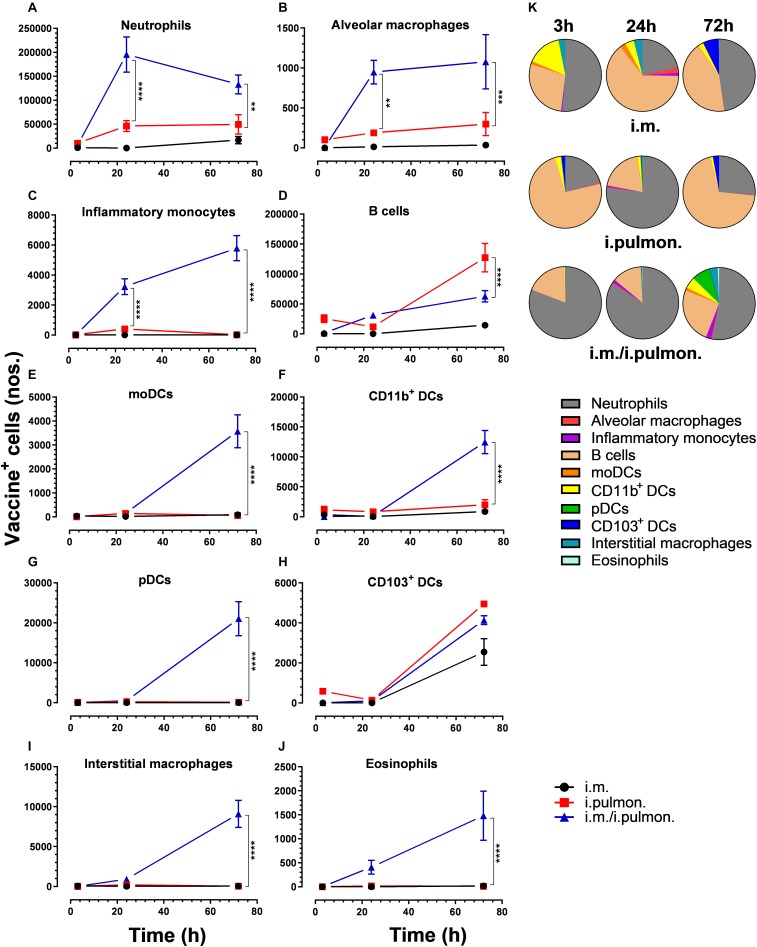
Airway mucosal pull immunization after parenteral immunization with H56/CAF01 increases vaccine uptake by innate myeloid cells in the lungs as compared to parenteral or airway mucosal priming alone. BALB/c mice were immunized with Alexa Fluor^®^ 647-labeled H56/DiR-labeled CAF01 *via* the i.m. or i.pulmon. or i.m. – i.pulmon. routes, and the vaccine uptake by lung cells was assessed by flow cytometry 3, 24, and 72 h post-immunization. Numbers of vaccine^+^ (H56^+^/CAF01^+^) **(A)** neutrophils (Ly6G^+^), **(B)** alveolar macrophages (F4/80^+^CD11b^–^ ), **(C)** inflammatory monocytes (Ly6C^+^CD11b^+^), **(D)** B cells (CD19^+^), **(E)** moDCs (CD11c^+^F4/80^+^CD11b^+^CD64^+^), **(F)** CD11b^+^ DCs (CD11c^+^CD11b^+^), **(G)** pDCs (CD11c^+^Ly6C^+^F4/80^–^ CD11b^–^ ), **(H)** CD103^+^ DCs (CD11c^+^CD11b^–^ CD103^+^), **(I)** interstitial macrophages (F4/80^+^CD11b^+^), and **(J)** eosinophils (SiglecF^+^) in the lungs. **(K)** Fraction of vaccine^+^ (H56^+^/CAF01^+^) cells in the lungs at 3, 24, and 72 h post-immunization. Data points represent *n* = 4, and they display mean values ± SEM. **p < 0.01, ***p < 0.001, ****p < 0.0001 vs. i.pulmon. immunization *via* two-way ANOVA with Tukey’s post-test.

**FIGURE 2:**
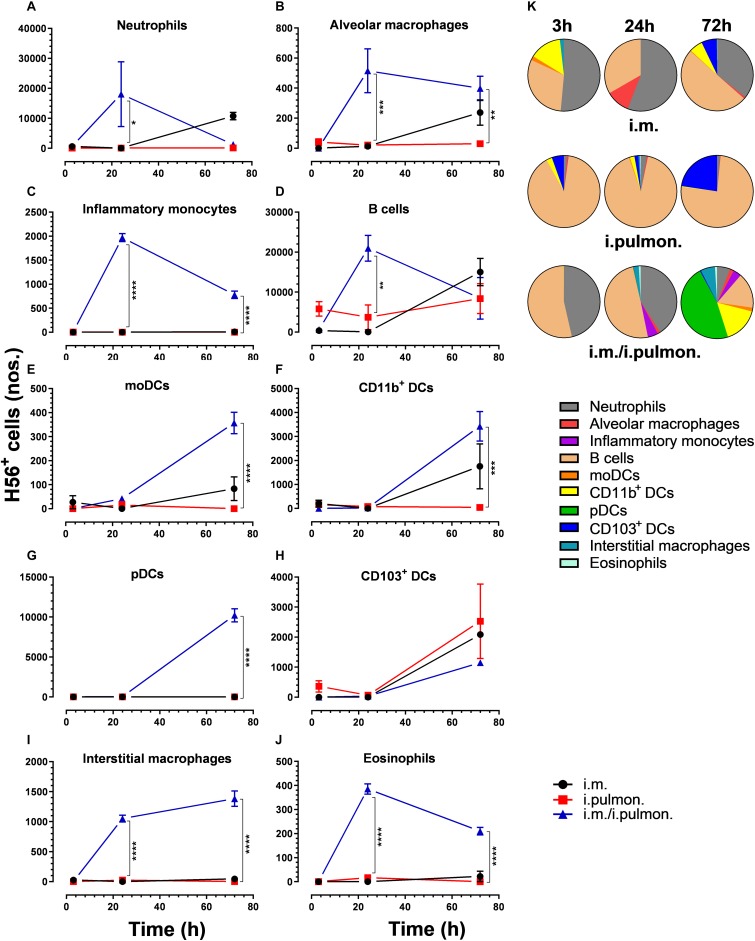
H56 uptake by different immune cells in the lungs following prime or prime – pull immunization with H56/CAF01. BALB/c mice were immunized with Alexa Fluor^®^ 647-labeled H56/DiR-labeled CAF01 *via* i.m. or i.pulmon. or i.m. – i.pulmon. routes, and the H56^+^ uptake by lung cells was assessed by flow cytometry 3, 24, and 72 h post-immunization. Numbers of H56^+^ **(A)** neutrophils (Ly6G^+^), **(B)** alveolar macrophages (F4/80^+^CD11b^–^ ), **(C)** inflammatory monocytes (Ly6C^+^CD11b^+^), **(D)** B cells (CD19^+^), **(E)** moDCs (CD11c^+^F4/80^+^CD11b^+^CD64^+^), **(F)** CD11b^+^ DCs (CD11c^+^CD11b^+^), **(G)** pDCs (CD11c^+^Ly6C^+^F4/80^–^ CD11b^–^ ), **(H)** CD103^+^ DCs (CD11c^+^CD11b^–^ CD103^+^), **(I)** interstitial macrophages (F4/80^+^CD11b^+^), and **(J)** eosinophils (SiglecF^+^) in the lungs. **(K)** Fraction of H56^+^ cells in the lungs at 3, 24 and 72 h post-immunization. Data points represent *n* = 2, and they display mean values ± SEM. *p < 0.05, **p < 0.01, ***p < 0.001, ****p < 0.0001 vs. i.pulmon. immunization *via* two-way ANOVA with Tukey’s post-test.

Similar to our previous study using an i.m. prime – i.pulmon. pull immunization strategy for the H56/CAF01 vaccine, which induced strong lung mucosal CD4^+^ T-cell immunity (24), mice were primed once by i.m. immunization followed by i.pulmon. pull immunization. The cellular uptake of Alexa Fluor^®^-labeled H56/DiR-labeled CAF01 in the lungs was evaluated by multicolor flow cytometry 3, 24, and 72 h post-immunization and compared with the cellular uptake after i.pulmon. and i.m. priming immunizations alone (Supplementary Figure S1). The fluorescent labeling of CAF01 and its cellular uptake using flow cytometry was performed as previously reported (33). H56 was commercially labeled with Alexa Fluor^®^ 647 as previously reported for ovalbumin (33) and the fluorescent labeling did not influence the physicochemical properties of H56 (data not shown). Moreover, we have previously shown that radiolabeling of H56 did not influence its physicochemical properties (24). In general, we observed that at 72 h, i.pulmon. pull immunization induced a significantly higher vaccine uptake by immune cells in the lungs as compared to i.pulmon. priming immunization alone (Figure 1 and Supplementary Table S2). As expected, we did not observe any vaccine^+^ cells in the lungs after i.m. priming alone. In addition, there was a rapid influx of neutrophils into the lungs within the first 24 h after i.pulmon. pull immunization, and the number of neutrophils was significantly higher than the number of neutrophils detected at 24 and 72 h post-immunization in the lungs of mice only primed by i.pulmon. immunization (Figure 1A). A comparable trend was observed for alveolar macrophages (Figure 1B) and inflammatory monocytes (Figure 1C). At 72 h, a significantly higher number of vaccine^+^ B cells was detected following i.pulmon. priming as compared to i.pulmon. pull immunization (Figure 1D). Among the studied DC subsets, vaccine^+^ monocyte-derived DCs (moDCs) (Figure 1E), CD11b^+^ DCs (Figure 1F), and plasmacytoid DCs (pDCs) (Figure 1G) were detected at significantly higher numbers 72 h after i.pulmon. pull immunization than after i.pulmon. priming immunization alone, whereas there was no statistical significant difference in the number of CD103^+^ DCs at this time point (Figure 1H). Similarly, at 72 h, the numbers of vaccine^+^ interstitial macrophages (Figure 1I) and eosinophils (Figure 1J) were highest following i.pulmon. pull immunization. We also assessed the total fraction of each cell subset in the lungs 3, 24, and 72 h post-immunization (Figure 1K), though none of the subpopulation changes were different between groups. We did observe a non-significant trend that the neutrophils and the B cell population were the most abundant cell subsets in the lungs at the designated time points following the different immunization regimens. The CD11b^+^ DCs constituted a major fraction of the vaccine^+^ cell subsets, whereas the CD103^+^ DCs only made up a minor fraction, in particular at 3 and 72 h following i.m. or i.pulmon. immunization. The vaccine was also associated with pDCs, but vaccine association with this cell subset was only detectable at 72 h after i.pulmon. pull immunization. Evaluation of the H56^+^ uptake by the immune cells in the lungs (Figure 2) showed almost similar trends as the vaccine (H56/CAF01) uptake, except that the numbers of cells taking up the vaccine were 1.5–7.5 times higher than the numbers of cells displaying detectable H56 uptake. Another major difference was that the majority of the cellular subsets had taken up H56 as early as 24 h post-immunization as compared to 72 h in case of H56/CAF01. In general, the number of vaccine^+^ and H56^+^ cells were higher for the group vaccinated using the i.m. prime – i.pulmon. strategy as compared to the groups only vaccinated by prime immunization, which suggests activation of both innate and memory responses.

### H56/CAF01 Parenteral Prime-Airway Mucosal Pull Immunization Enhances Vaccine Uptake by Lung Endothelial Cells and Type I Epithelial Cells as Compared to Parenteral or Airway Mucosal Prime Immunization Alone

**FIGURE 3:**
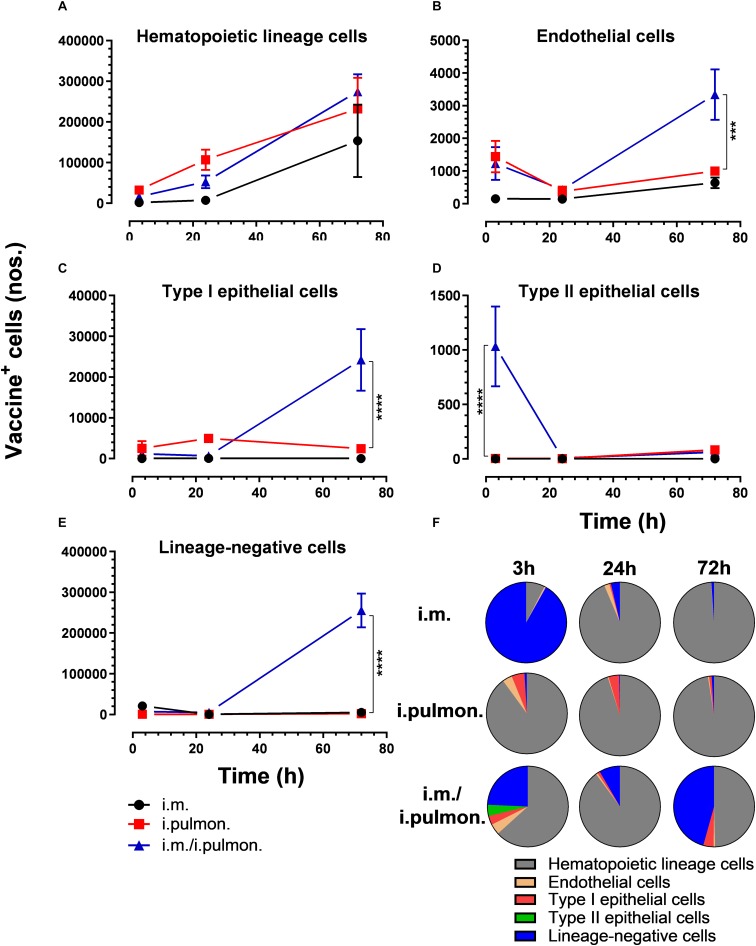
Intramuscular (i.m.) prime-intrapulmonary (i.pulmon.) pull immunization with H56/CAF01 increases vaccine uptake by lung endothelial cells and type I epithelial cells as compared to either i.m. or i.pulmon. immunization alone. BALB/c mice were immunized with Alexa Fluor-labeled H56/DiR-labeled CAF01 *via* the i.m. or i.pulmon. or i.m. – i.pulmon. routes, and vaccine uptake by lung cells was assessed by flow cytometry 3, 24, and 72 h post-immunization. Numbers of vaccine^+^ (H56^+^/CAF01^+^) **(A)** hematopoietic lineage cells (CD45^+^CD31^–^ CD326^–^ ), **(B)** endothelial cells (CD45^–^ CD31^+^CD326^–^ ), **(C)** type I epithelial cells (CD45^–^ CD31^–^ CD326^+^CD74^–^ Podoplanin^+^), **(D)** type II epithelial cells (CD45^–^ CD31^–^ CD326^+^CD74^+^Podoplanin^–^ ), and **(E)** lineage-negative cells (CD45^–^ CD31^–^ CD326^–^ ) in the lungs. **(F)** Fraction of vaccine^+^ (H56^+^/CAF01^+^) cells in the lungs at 3, 24, and 72 h post-immunization. Data points represent mean values ± SEM (*n* = 4). ***p < 0.001, ****p < 0.0001 vs. i.pulmon. immunization *via* two-way ANOVA with Tukey’s post-test.

**FIGURE 4:**
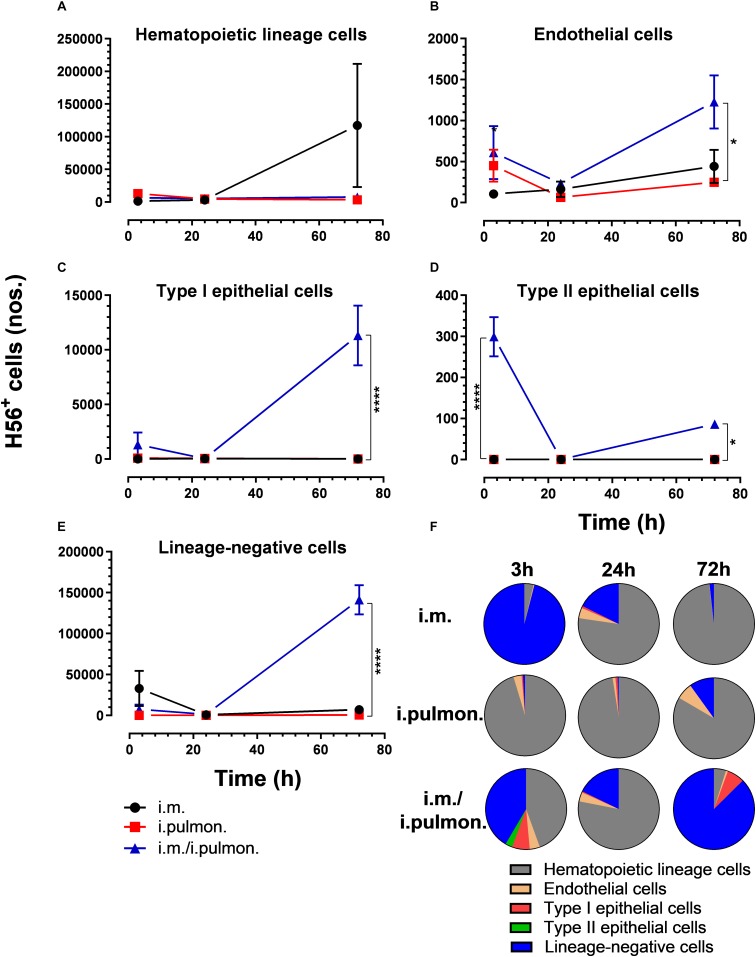
H56 uptake by epithelial cells, endothelial cells, hematopoietic lineage cells, and lineage-negative cells in the lungs following prime or prime – pull immunization with H56/CAF01. BALB/c mice were immunized with Alexa Fluor^®^ 647-labeled H56/DiR-labeled CAF01 *via* the i.m. or i.pulmon. or i.m. – i.pulmon. routes, and the H56^+^ uptake by lung cells was assessed by flow cytometry 3, 24 and 72 h post-immunization. Numbers of vaccine^+^ (H56^+^/CAF01^+^) **(A)** hematopoietic lineage cells (CD45^+^CD31^–^ CD326^–^ ), **(B)** endothelial cells (CD45^–^ CD31^+^CD326^–^ ), **(C)** type I epithelial cells (CD45^–^ CD31^–^ CD326^+^CD74^–^ Podoplanin^+^), **(D)** type II epithelial cells (CD45^–^ CD31^–^ CD326^+^CD74^+^Podoplanin^–^ ), and **(E)** lineage-negative cells (CD45^–^ CD31^–^ CD326^–^ ) in the lungs. **(F)** Fraction of H56^+^ cells in the lungs at 3, 24, and 72 h post-immunization. Data points represent *n* = 2 and display mean values ± SEM. *p < 0.05, ****p < 0.0001 vs. i.pulmon. immunization *via* two-way ANOVA with Tukey’s post-test.

We also evaluated the uptake of the fluorescently labeled H56/CAF01 vaccine by pulmonary epithelial cells, endothelial cells, hematopoietic lineage cells, and lineage-negative cells (Supplementary Figure S2). No significant differences in the vaccine uptake by hematopoietic lineage cells could be measured between the i.pulmon. prime versus i.pulmon. pull immunization (Figure 3A). At 3 and 24 h post-immunization, no significant differences were observed in the vaccine uptake by the majority of the lung cell populations following i.pulmon. priming or i.pulmon. pull immunization (Figures 3B–E). However, the number of vaccine^+^ type II epithelial cells was significantly higher following i.pulmon. pull immunization than i.pulmon. priming immunization alone (Figure 3D). At 72 h, a significantly higher number of vaccine^+^ endothelial cells (Figure 3B), type I epithelial cells (Figure 3C), and lineage-negative cells (Figure 3E) were measured following i.pulmon. pull as compared to the number of cells after i.pulmon. prime immunization alone. Evaluation of the total fraction of these pulmonary cell populations in the lungs (Figure 3F) did not show any significant differences among the groups. The data showed a non-significant trend that the hematopoietic cells were largely the dominant cell subsets in the lungs at the examined time points when applying different immunization regimens. Endothelial cells and type I epithelial cells constituted the other fraction of vaccine^+^ cell subsets. The vaccine^+^ lineage-negative cells comprised the dominant fraction of cells 3 h after i.m. immunization, and this was observed at all time points following the i.m prime – i.pulmon. pull immunization. We also evaluated the H56 uptake by the endothelial cells and epithelial cells in the lungs (Figure 4) and found an almost similar cellular distribution as for the H56/CAF01 uptake. However, the numbers of cells taking up the vaccine (H56 + CAF01) were in general 2–3.75 times higher than the number of cells that displayed detectable levels of H56 uptake.

### Differential Splenic Cellular Pharmacokinetics of H56/CAF01 Upon Prime and Prime-Pull Immunization

**FIGURE 5:**
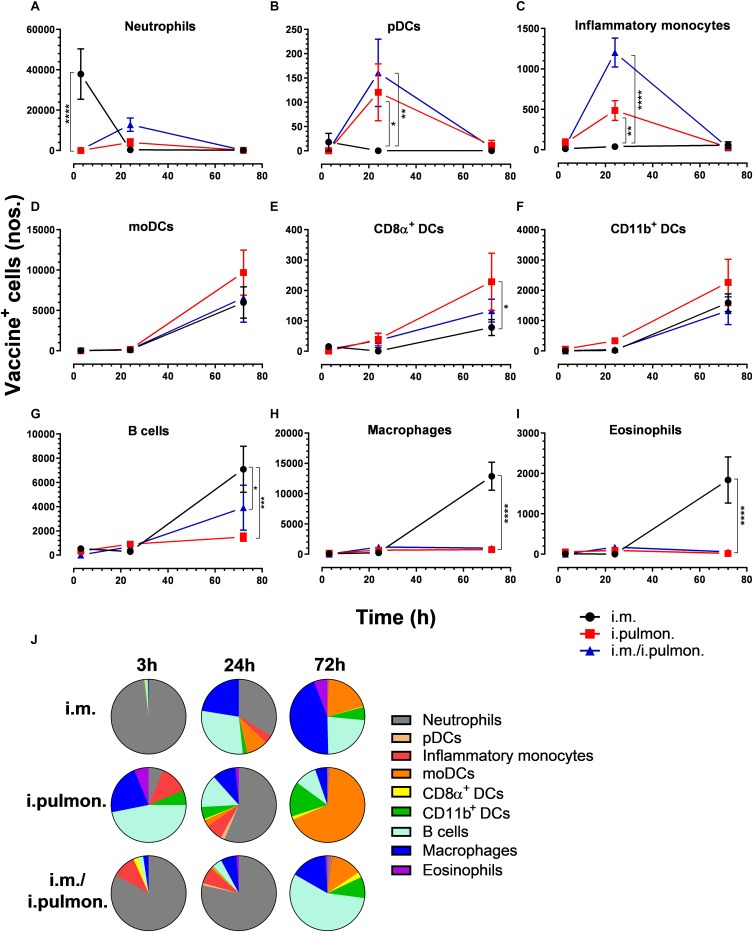
Differential vaccine uptake by innate myeloid and antigen-presenting cells following parenteral or mucosal prime and parenteral prime-mucosal pull immunization with H56/CAF01. BALB/c mice were immunized with Alexa Fluor^®^-labeled H56/DiR-labeled CAF01 *via* the i.m. or i.pulmon. or i.m. – i.pulmon. routes, and the vaccine uptake by spleen cells was assessed by flow cytometry 3, 24, and 72 h post-immunization. Numbers of vaccine^+^ (H56^+^/CAF01^+^) **(A)** neutrophils (Ly6G^+^), **(B)** pDCs (CD11c^+^Ly6C^+^F4/80^–^ CD11b^–^ ), **(C)** inflammatory monocytes (Ly6C^+^CD11b^+^), **(D)** moDCs (CD11c^+^F4/80^+^CD11b^+^CD64^+^), **(E)** CD8α^+^ DCs (CD11c^+^CD11b^–^ CD8α ^+^), **(F)** CD11b^+^ DCs (CD11c^+^CD11b^+^), **(G)** B cells (CD19^+^), **(H)** macrophages (F4/80^+^CD11b^+^), and **(I)** eosinophils (SiglecF^+^) in the spleen. **(J)** Fraction of vaccine^+^ (H56^+^/CAF01^+^) cells in the spleen at 3, 24, and 72 h post-immunization. Data points represent *n* = 4, and they display mean values ± SEM. *p < 0.05, **p < 0.01, ***p < 0.001, ****p < 0.0001 vs. i.m. immunization *via* two-way ANOVA with Tukey’s post-test.

**FIGURE 6:**
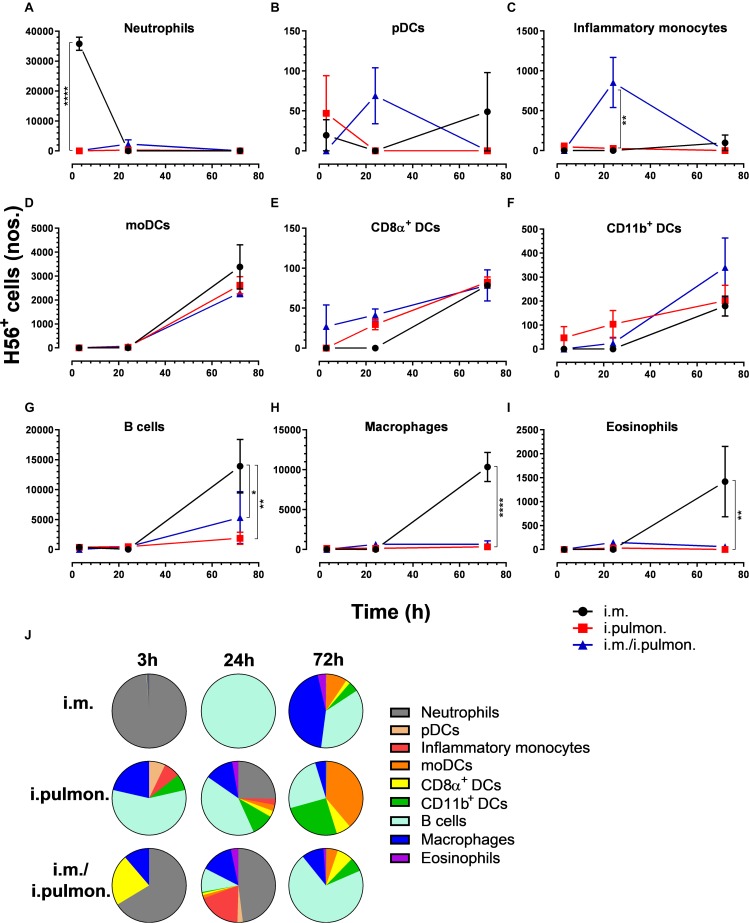
H56 uptake by splenic cells following parenteral or airway mucosal prime or parenteral prime – airway mucosal pull immunization with H56/CAF01. BALB/c mice were immunized with Alexa Fluor^®^ 647-labeled H56/DiR-labeled CAF01 *via* i.m. or i.pulmon. or i.m. – i.pulmon. routes, and the H56^+^ uptake by spleen cells was assessed by flow cytometry 3, 24, and 72 h post-immunization. Numbers of **(A)** neutrophils (Ly6G^+^), **(B)** pDCs (CD11c^+^Ly6C^+^F4/80^–^ CD11b^–^ ), **(C)** inflammatory monocytes (Ly6C^+^CD11b^+^), **(D)** moDCs (CD11c^+^F4/80^+^CD11b^+^CD64^+^), **(E)** CD8α^+^ DCs (CD11c^+^CD11b^–^ CD8α ^+^), **(F)** CD11b^+^ DCs (CD11c^+^CD11b^+^), **(G)** B cells (CD19^+^), **(H)** macrophages (F4/80^+^CD11b^+^), and **(I)** eosinophils (SiglecF^+^) in the spleen. **(J)** Fraction of H56^+^ cells in the spleen at 3, 24, and 72 h post-immunization. Data points represent *n* = 2, and they display mean values ± SEM. *p < 0.05, **p < 0.01, ****p < 0.0001 vs. i.m. immunization *via* two-way ANOVA with Tukey’s post-test.

We also assessed the cellular uptake of the H56/CAF01 vaccine by innate myeloid cells and APCs in the spleen (Supplementary Figure S3). Overall, we found differences in the vaccine uptake by splenocyte populations when applying the three different immunization strategies (Figure 5 and Supplementary Table S2). At 3 h post-i.m. immunization, a significantly higher number of vaccine^+^ neutrophils was observed as compared to mucosal immunizations (Figure 5A). At 24 h, significantly higher numbers of pDCs (Figure 5B) and inflammatory monocytes (Figure 5C) were observed after i.pulmon. and i.m. – i.pulmon. immunization as compared to i.m. immunization alone. No other differences were observed at 3 and 24 h among the different immunization strategies. At 72 h, the vaccine^+^ moDCs (Figure 5D), CD8α^+^ DCs (Figure 5E), and CD11b^+^ DCs (Figure 5F) were higher for all immunization groups than at 3 and 24 h, but were not different among the immunization strategies used. A significantly higher number of vaccine^+^ CD8α^+^ DCs were observed following i.pulmon. immunization as compared to the i.m. immunization. The numbers of vaccine^+^ neutrophils (Figure 5A), pDCs (Figure 5B), and inflammatory monocytes (Figure 5C) at 72 h decreased, as compared to the number of vaccine^+^ cells measured 24 h post-immunization, and the numbers of cells were not different among the groups. For mice vaccinated by i.m. immunization, significantly higher numbers of vaccine^+^ B cells (Figure 5G), macrophages (Figure 5H), and eosinophils (Figure 5I) were detected, as compared to the numbers of cells measured after i.pulmon. prime or pull immunization at 72 h. Among the total fraction of vaccine^+^ splenic cell types at 3, 24, and 72 h post-immunization (Figure 5J), we did not observe any significant differences between the groups. However, the percentage subpopulations of all the H56^+^ splenic cells (Figure 6J) were statistically higher (*p* < 0.05) following 24 h of i.m./i.pulmon. immunization as compared to i.m. immunization. We did observe a non-significant trend that the neutrophils, macrophages and B cells were the predominant cell populations taking up the vaccine, independently of the immunization strategy. The CD11b^+^ DCs and moDCs constituted the dominant DC subsets, in particular at 72 h post-immunization. In general, there was a diversified participation of innate myeloid cells and APCs in the vaccine uptake in the spleen following i.pulmon. immunization. For the H56^+^ uptake (Figure 6), almost similar trends were apparent, as compared to the vaccine uptake, but the numbers of cells taking up the vaccine were 1.2-8 times higher than the numbers of cells displaying detectable H56 uptake.

### Mucosal Pull Immunization of Mice Parenterally Primed With H56/CAF01 Promotes Upregulation of CD86 Expression by Dendritic Cells in the Lung-Draining Lymph Nodes as Compared to Parenteral or Mucosal Prime Immunization Alone

**FIGURE 7:**
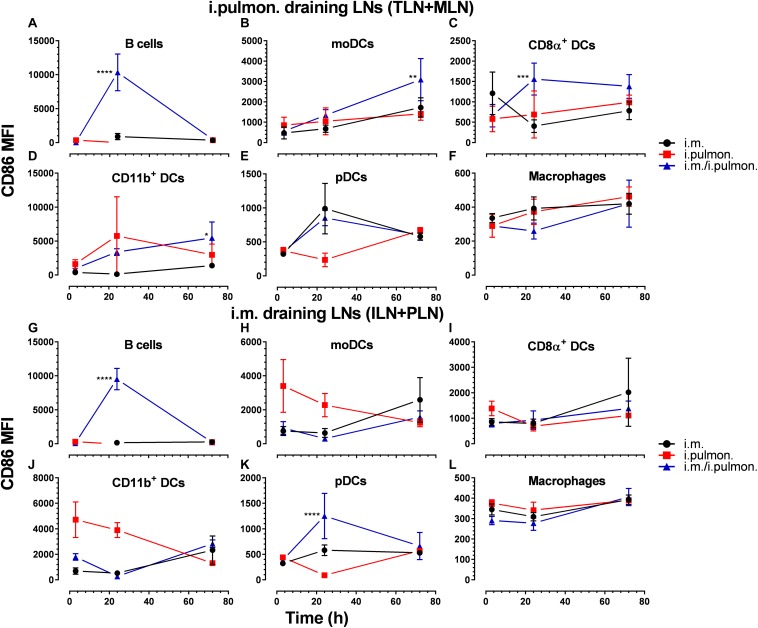
The expression of CD86 by dendritic cells in the lung-draining lymph nodes is upregulated after parenteral prime and mucosal pull immunization of mice with H56/CAF01, as compared to the CD86 levels in mice vaccinated by parenteral or mucosal prime immunization alone. BALB/c mice were immunized with Alexa Fluor^®^-labeled H56/DiR-labeled CAF01 *via* the i.m. or i.pulmon. or i.m. – i.pulmon. routes, and the vaccine uptake in the lymph nodes draining the i.m. administration site [inguinal (ILN) and popliteal (PLN)] or the i.pulmon. administration site [tracheobronchial (TLN) and mediastinal (MLN)] was assessed by flow cytometry 3, 24, and 72 h post-immunization. The relative surface expression of CD86 by antigen-presenting cells was assessed and expressed as mean fluorescence intensity (MFI). CD86 surface expression by vaccine^+^ (H56^+^/CAF01^+^) **(A)** B cells (CD19^+^), **(B)** moDCs (CD11c^+^F4/80^+^CD11b^+^CD64^+^), **(C)** CD8α^+^ DCs (CD11c^+^CD11b^–^ CD8α^+^), **(D)** CD11b^+^ DCs (CD11c^+^CD11b^+^), **(E)** pDCs (CD11c^+^Ly6C^+^F4/80^–^ CD11b^–^ ), and **(F)** macrophages (F4/80^+^CD11b^+^) in the TLN and MLN, and **(G)** B cells (CD19^+^), **(H)** moDCs (CD11c^+^F4/80^+^CD11b^+^CD64^+^), **(I)** CD8α^+^ DCs (CD11c^+^CD11b^–^ CD8α^+^), **(J)** CD11b^+^ DCs (CD11c^+^CD11b^+^), **(K)** pDCs (CD11c^+^Ly6C^+^F4/80^–^ CD11b^–^ ), and **(L)** macrophages (F4/80^+^CD11b^+^) in the ILN and PLN at 3, 24, and 72 h post-immunization. Data points represent *n* = 4, and they display mean values ± SEM. *p < 0.05, **p < 0.01, ***p < 0.001, ****p < 0.0001 vs. i.m. immunization *via* two-way ANOVA with Tukey’s post-test.

Clear differences were observed in the vaccine uptake by immune cells in the lungs of mice vaccinated using different immunization strategies. Therefore, we investigated if differences in the draining lymph node innate environment after mucosal pull immunization may contribute to the differences measured in the vaccine uptake when using the three immunization strategies (Supplementary Figure S3) and to the higher cell-mediated and humoral immune responses following i.m. prime – i.pulmon. pull immunization, as reported previously (24). We found that the activation states, assessed as the CD86 surface expression by B cells (at 24 h, Figure 7A), moDCs (at 72 h, Figure 7B), CD8α^+^ DCs (at 24 h, Figure 7C), and CD11b^+^ DCs (at 72 h, Figure 7D) in the tracheobronchial lymph nodes (TLNs) and mediastinal lymph nodes (MLN) draining the lungs in i.m. prime and i.pulmon. pull immunized mice, were significantly higher than the activation states after i.m. or i.pulmon. immunization alone. There were no differences between the immunization strategies in the CD86 surface expression by pDCs (Figure 7E) and macrophages (Figure 7F) in the TLNs and MLNs. Interestingly, B cells (Figure 7G) and pDCs (Figure 7K) were activated distal to the site of immunization in the inguinal lymph nodes (ILNs) and the popliteal lymph nodes (PLNs) at 24 h post-prime-pull immunization, and only DCs showed increased activation after i.m. immunization. However, there was no difference in the surface expression of CD86 between the immunization strategies in moDCs (Figure 7H), CD8α^+^ DCs (Figure 7I), CD11β^+^ DCs (Figure 7J), and macrophages (Figure 7L). Overall, there was an increased activation state of vaccine^+^ DCs in the lymph nodes draining the lungs after i.m. prime – i.pulmon. pull immunization with H56/CAF01.

### A Uniform DDA Distribution and Signal Intensity in the Lungs Can Be Detected for at Least 2 Weeks After Intrapulmonary Administration of CAF01

**FIGURE 8:**
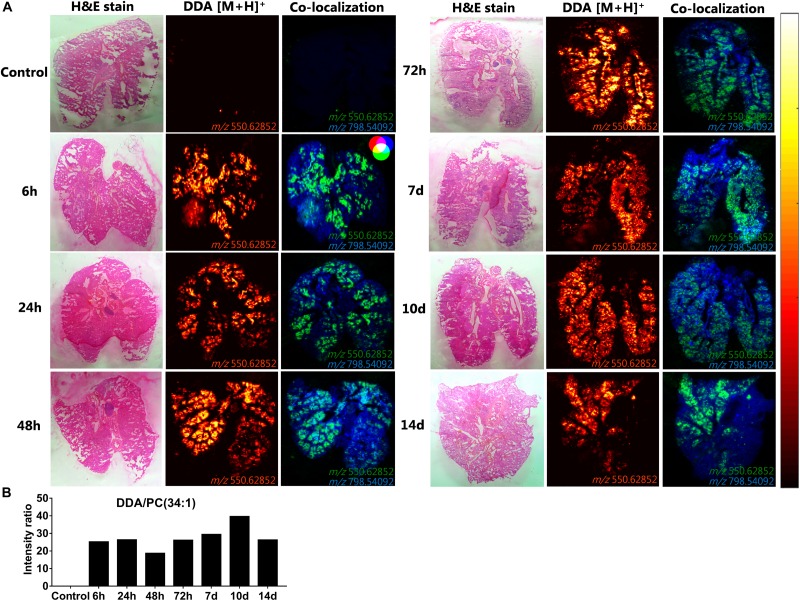
Uniform distribution and signal intensity of dimethyldioctadecylammonium (DDA) bromide in cryo-sections of lungs of mice, which have been dosed intrapulmonary (i.pulmon.) with CAF01. BALB/c mice were immunized once with CAF01 *via* the i.pulmon. route, and matrix-assisted laser desorption/ionization mass spectrometry imaging (MALDI-MSI) was performed on lung cryo-sections at 6, 24, 48, and 72 h, and 7, 10, and 14 days after the immunization. Untreated mice served as negative control. **(A)** Hematoxylin and eosin (H&E) staining (left panels), MALDI-MSI-based distribution of DDA [M + H]^+^ (m/z 550.629 ± 0.002, middle panels), and mass spectrometry (MS) co-localization images (right panels) of DDA [M + H]^+^ (green) and PC (34:1) [M + K]^+^ (m/z 798.541 ± 0.002) (blue) in the lungs at different time points after i.pulmon. administration of CAF01. **(B)** Signal intensity ratios between DDA and PC (34:1) at different time points of the study, which were calculated after drawing a region of interest (ROI) across the lung sections and comparing the MS signal intensities in the respective ROIs. All images were measured in the positive ion mode by MALDI-MSI at a pixel size of 100 μm.

To investigate the spatiotemporal distribution of the CAF01 constituent lipids (DDA and TDB) in the lungs, MALDI-MSI was performed on cryo-sections of lung tissue isolated 6, 24, 48, and 72 h, and 7, 10, and 14 days after i.pulmon. administration of CAF01 to mice, and we compared the results with the signals obtained from cryo-sections of lungs from naïve mice. At these specific time points, we then compared the signal intensity ratios between DDA and TDB, respectively, and the endogenous lipid PC (34:1) in the positive ion mode in a semi-quantitative way. We found that DDA (m/z 550.62) could be detected at all examined time points after i.pulmon. administration of CAF01 (Figure 8). In addition, DDA displayed a homogeneous tissue distribution in the lungs at all examined time points at comparable signal intensities. Lung cryo-sections from negative control mice did not display any detectable MS signals of DDA and TDB. Ionized PC (34:1) (m/z 798.541) was also uniformly expressed in the lung cryo-sections at all investigated time points (Supplementary Figure S4). Co-localization analysis showed that DDA and PC (34:1) were present together in the lung sections (Figure 8A), and the signal intensity ratio of DDA and PC (34:1) appeared rather constant throughout the study (Figure 8B). As compared to DDA, the signal intensity for TDB (m/z 1025.726) was relatively lower, and the signal was apparently not distributed homogeneously in the lung sections (Supplementary Figure S5A). The signal intensity ratio of TDB relative to PC (34:1) increased from 6 h and reached a maximum at 48 h post-administration, after which the intensity ratio remained low until day 14, where the TDB signal was no longer detectable (Supplementary Figure S5B).

### Increased BMP Lipids in Alveloar Macrophages Following Intrapulmonary Administration of CAF01 Is Suggestive of Altered Phagocytic Activity

**FIGURE 9:**
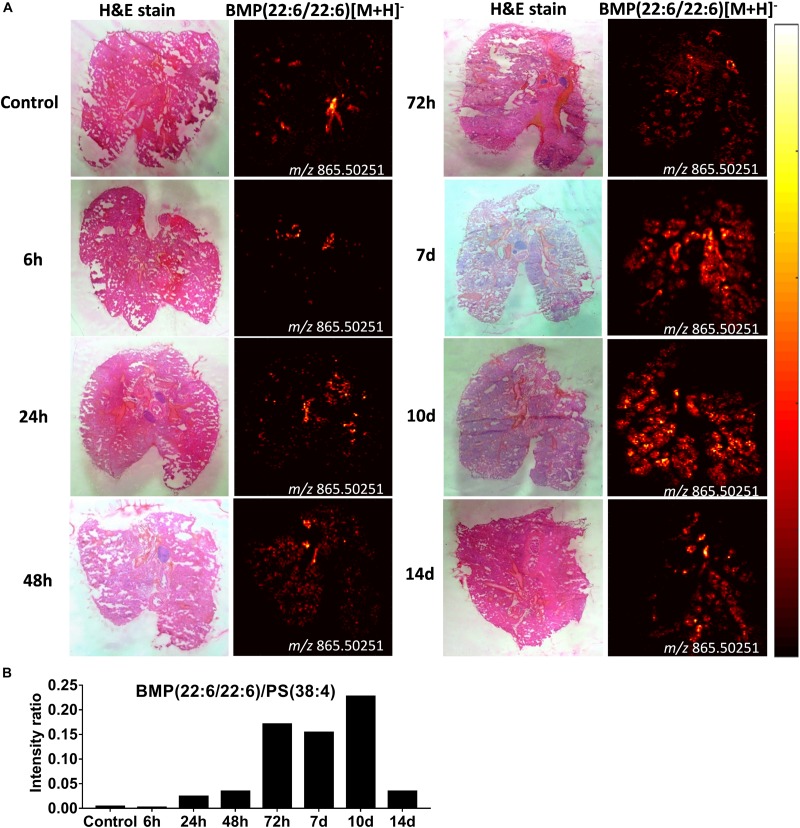
Increased bis(monoacylglycero)phosphate (BMP) expression in the lungs of mice dosed intrapulmonary (i.pulmon.) with CAF01. BALB/c mice were immunized once with CAF01 *via* the i.pulmon. route, and matrix-assisted laser desorption/ionization mass spectrometry imaging (MALDI-MSI) was performed on lung cryo-sections collected 6, 24, 48, 72 h, and 7, 10, and 14 days after immunization. Untreated mice served as negative control. **(A)** Hematoxylin and eosin (H&E) staining (left panels) and MALDI-MSI-based distribution of BMP (22:6/22:6) [M + H]^–^ (m/z 865.503, right panels) in cryo-sections of lungs isolated at different time points after i.pulmon. dosing with CAF01. **(B)** Signal intensity ratios between BMP and PS (38:4) [M + H]^–^ (m/z 810.529 ± 0.002) at different time points of the study, which were calculated by drawing a region of interest (ROI) across the lung sections and comparing the MS signal intensities in the respective ROIs. All images were measured in the negative ion mode by using MALDI-MSI at a pixel size of 100 μm.

From the same imaging experiments, images were generated of selected biomarker phospholipids, i.e., BMP and lysoPC, and sphingolipids, i.e., ceramides, in the lungs after i.pulmon. administration of CAF01. BMP is a recognized biomarker of phagocytozing macrophages (34), and it is abundantly expressed in the late endosomes and lysosomes of alveolar macrophages (35), while lysoPC (36) and ceramides (37) are known biomarkers of inflammation. We observed that 6 h after i.pulmon. administration of CAF01, the BMP (22:6/22:6, m/z 865.503) expression level in the lungs was comparable to the BMP level measured for control animals (Figure 9A). However, from 24 h and onward, the BMP signal increased consistently reaching a maximum at day 10 of the study, after which it was decreased at day 14. The ionized PS (38:4) (m/z 810.529), which is the most abundant phosphatidylserine species in the lungs (38), was expressed in the lung sections at all studied time points (Supplementary Figure S6). Analysis of the signal intensity ratio of BMP (22:6/22:6) as compared to PS (38:4) (m/z 810.529) showed that the intensity ratio of BMP/PS was increased from 24 h post-administration, reached a threshold at day 10 of the study, after which it was decreased at day 14 (Figure 9B). The increased level of BMP after i.pulmon. administration of CAF01 therefore suggests an altered phagocytic activity in alveolar macrophages. We did not observe any expression of ceramides and cholesteryl esters at the investigated time points after administration of CAF01 (data not shown). The phospholipid LysoPC (16:0) (m/z 518.323) was primarily distributed homogeneously in the lungs following CAF01 administration (Supplementary Figure S7A). Analysis of the signal intensity ratio of LysoPC (16:0)/PC (34:1) confirmed this observation. The intensity ratio of LysoPC/PC after administration of CAF01 remained low until day 10, or at the same level at day 14, compared to negative control lungs (Supplementary Figure S7B). This data therefore suggests that i.pulmon. administration of CAF01 does not induce any apparent inflammation or tissue damage in the lungs because the LysoPC and ceramides levels are not influenced by CAF01 administration.

## Discussion

Here, we investigated further our previous finding that parenteral prime and airway mucosal pull immunization with the H56/CAF01 vaccine induces higher humoral and cell-mediated immune responses than parenteral immunization alone (24) by providing a cellular basis that may explain the enhanced immunogenicity. We show that the i.m. prime – i.pulmon. pull immunization regimen with H56/CAF01 induces a higher vaccine uptake by pulmonary APCs, endothelial cells, and type I epithelial cells as well as splenic inflammatory macrophages as compared to the vaccine uptake measured after i.m. and i.pulmon. immunization, respectively. In addition, there is an increased activation state of vaccine^+^ DCs and B cells in the lung- and intramuscular-draining lymph nodes following i.m. prime – i.pulmon. pull immunization with H56/CAF01, as compared to the activation state measured after immunization using the i.m. and i.pulmon. routes of administration, respectively. A major difference between the evaluated immunization strategies is the activation of innate immunity after i.m. and i.pulmon. prime immunization alone, as compared to the activation of both innate and memory immune responses after i.m. prime – i.pulmon pull immunization, in particular at the 24 h time point of the study. Importantly, we show for the first time that MSI is a useful tool to investigate the biodistribution of a lipid-based vaccine adjuvant. We report that following pulmonary immunization, CAF01 is homogeneously distributed in the lung parenchyma and is present in the lungs for at least 2 weeks without inducing any measurable local tissue damage or inflammation. We further report an increased BMP signal in the lungs following immunization, which in ischemic brain tissue has been shown to correlate with an increased phagocytic activity of cerebral macrophages (34). Hence, the increased BMP activity measured in the lungs after administration of CAF01 may be correlated with an altered phagocytic activity in alveolar macrophages.

The lungs are highly vascularized with a large surface area and an extensive network of immune cells surveilling the mucosa against microbial attacks, which make lung tissue an attractive target for the administration of subunit vaccine antigens and immunopotentiating adjuvants (39). Subunit vaccines may elicit immunopotentiating effects by interacting with the highly specialized network of immune cells, e.g., DCs and macrophages that capture the vaccines via pattern-recognition receptors and transport them (antigen + adjuvant) to the regional lymph nodes, where antigen-specific T cell activation takes place (14). T cell-mediated protection against *Mtb* is recognized to be dependent on the ability of the antigen-specific T-cells to home back to the lung parenchyma and directly interact with infected cells (40, 41). Accordingly, a number of airway mucosal immunization strategies, based on either mucosal BCG vaccination or heterologous mucosal vaccination with viral vectors encoding *Mtb* antigens following BCG priming, have been tested and found to enhance the number of protective lung-resident T cells against TB (42–44). Hence, pulmonary delivery of subunit vaccines represents an attractive strategy for inducing antigen-specific T cell immunity in the lungs (39, 45). We envisage to use this strategy for immunization of adolescents and adults with H56/CAF01 vaccine. BCG is administered to newborns immediately after birth and has over 70% protective efficacy against tuberculous meningitis and miliary TB (46), while in adults, BCG vaccination fails to completely protect against pulmonary TB and has a very variable protective efficacy (0–80%) (47). BCG vaccination also reduces mortality in newborn and children because of non-specific cross-protection induced by this vaccine against other unrelated pathogens (48). Therefore, keeping in view the beneficial effects of BCG vaccination in children, our long term strategy is to boost the BCG-primed immune responses (in infants and neonates) with parenteral prime and mucosal pull immunization of H56/CAF01 vaccine (in adolescents and adults).

Recently, we demonstrated that parenteral prime and i.pulmon. pull immunization with the H56/CAF01 vaccine induces significantly higher airway mucosal as well as systemic IgA and polyfunctional CD4^+^ T cells as compared to parenteral prime and pull immunization (24). However, the cellular basis of this increased immune response after parenteral prime and mucosal pull immunization was unknown. The results of our current work provide strong evidence that this enhanced immunity may be by virtue of an increased vaccine uptake by pulmonary APCs, including DCs and macrophages, and/or an enhanced activation of DCs in the lung-draining lymph nodes. In the lungs, several DC subsets and macrophages reside possessing specialized functions with respect to antigen uptake, presentation and initiation of immune responses (49). In the steady state, the lungs are populated by two major subsets of conventional DCs (cDCs), i.e., CD103^+^ and CD11b^+^ cDCs, and pDCs, while moDCs migrate into the lung parenchyma in response to inflammation (28). In our study, the H56/CAF01 vaccine is taken up by respiratory tract APCs, but the extent of uptake is highly dependent on the specific immunization strategy. Pulmonary DCs and macrophages were strongly positive for H56/CAF01 administered by applying the parenteral prime – mucosal pull immunization strategy as compared to the H56/CAF01 levels measured after parenteral immunization alone.

Using virosomes and liposomes as delivery systems without or with conjugated ovalbumin, it has previously been shown that DCs and macrophages take up equally well nanoparticles administered intranasally (50). However, another study reported enhanced uptake of latex particles by alveolar macrophages, as compared to pulmonary DCs, after intranasal administration (51). Recently, airway mucosal pull immunization by H56/CAF01 immunization was demonstrated to induce significantly increased numbers and activation state of alveolar macrophages in the lungs (23). At the earliest time point investigated in the present study, i.e., 3 h after administration, our measurements did not show any detectable vaccine uptake in the airways or trafficking to the lung-draining LNs, as reported previously (12). Only 24 h after immunization, there were visible differences in the vaccine uptake between the different APCs, depending on the specific immunization strategy. We also observed a significantly higher number of vaccine^+^ neutrophils following mucosal pull immunization as compared to the number of vaccine^+^ neutrophils after single immunizations. Among the visceral organs, the lungs contain the highest proportion of neutrophils, which might facilitate the activation and differentiation of antigen-specific CD4^+^ T cells *via* cross-talk with DCs (52, 53). Therefore, our results show that there are distinct differences in the vaccine uptake by innate myeloid APCs and B cells, depending on the specific immunization strategy (prime versus prime – pull). Similarly, a high phenotypic diversity of neutrophils, monocytes, and DCs was observed between prime and pull immunization of cynomolgus macaques with the modified vaccinia virus Ankara (54).

In addition to professional APCs, a variety of other cell types may present antigens to T-helper cells, including epithelial cells (55, 56). Lung epithelial cells can present antigens, and they play an important role for the induction of local immune responses in the lungs (55, 57). We found that mucosal pull immunization induces a higher number of vaccine^+^ type II epithelial cells within 3 h and a higher number of type I epithelial cells at 72 h post-immunization as compared to parenteral immunization alone. Both type I (57) and II (58) epithelial cells possess the ability to present antigens, but they differ in their expression levels of major histocompatibility complex II molecules with higher expression levels in type II cells than in type I cells (57). We also observed lower major histocompatibility complex II expression in vaccine^+^ type I epithelial cells in this study (data not shown). The potential role of lung epithelial cells in antigen capture and presentation in immune responses is restricted to stimulate T cells previously presented to antigens by other APCs (59, 60). We found that the hematopoietic lineage and lineage-negative cells took up the vaccine, in particular at 72 h after immunization. Lineage-negative cells primarily include fibroblasts, smooth muscle cells and mesenchymal stem cells (26). The mesenchymal stem cells have the capability to capture and release antigens, which are subsequently captured by APCs (61).

The distinct differences in vaccine uptake by pulmonary immune cells after vaccine administration applying the three different immunization strategies correlated well with the subsequent activation state of DCs measured in the draining LNs. Previously, it has been shown that the activation state of CAF04- and CAF09-induced CD4^+^ and CD8^+^ T cells coincided with the activation of resident and migratory DCs in the spleen and the draining LNs after parenteral immunization (33). In the lung-draining LNs, resident CD8α^+^ and migratory CD11b^+^ DCs were significantly activated at 24 and 72 h after mucosal pull immunization, respectively, than after parenteral immunization. This increased activation state of vaccine^+^ DCs in the lung-draining lymph nodes (TLN + MLN) by i.m. prime – i.pulmon. pull immunization with H56/CAF01 supports our previous results where we observed that this immunization strategy induces high lung mucosal and systemic antibody and CD4^+^ responses (24). On the other hand, in the LNs draining the i.m. site of immunization (ILN + PLN), no significant differences between the activation state of CD8α^+^ and CD11b^+^ DCs were observed among the different immunization strategies used. Interestingly, B cells and pDCs are significantly activated in ILN + PLN at 24 h post-prime-pull immunization, and the fact that only DCs showed increased activation after i.m. immunization is supported by previous studies (62). Similarly, we found that all immunization strategies used for administering the H56/CAF01 vaccine promote differential cellular uptake by innate myeloid APCs present in the spleen. Our previous results show that i.m. prime – i.pulmon. pull immunization with H56/CAF01 induces highly comparable antibody and CD4^+^ responses in the spleen as the i.m. immunization (24). Overall, there were marked differences in the vaccine uptake by innate myeloid APCs, epithelial cells, and B cells and in the activation state of APCs between prime and prime – pull immunization with the H56/CAF01 vaccine. Recently, it was shown that the innate myeloid cells following the prime – pull with the modified vaccinia virus Ankara displayed higher activation states and enhanced expression of molecules involved in phagocytosis, antigen presentation, co-stimulation, chemotaxis, and inflammation (54). Recently, we showed that mucosal (intranasal) pull immunization of H56/CAF01 immunization significantly increased the early lung-localized vaccine T-cell response and increased early protection to a pulmonary *Mtb* challenge in mice (23). Therefore, our results demonstrating an improved antigen uptake and s stronger immune response following i.pulmon. pull immunization of H56/CAF01 immunization (24) are certainly promising for our efforts to develop a thermostable, dry powder-based H56/CAF01 vaccine intended for i.pulmon. immunization of BCG-primed individuals (63).

Mass spectrometry imaging can be used to identify the distribution and expression levels of molecules, e.g., biomarkers, peptides and proteins, and metabolites or drugs in tissue sections with high sensitivity and specificity, without the need to label the analyte (34, 64). This imaging technique can also be used to identify the spatiotemporal distribution of different phospholipids in various tissues, including the lungs (34, 65). Using SPECT-CT imaging, we have previously measured the pharmacokinetics of CAF01 for up to day 6 after pulmonary administration (24). However, there is still insufficient data on the long-term fate and safety of CAF01 administered in the respiratory tract. Here, we show using MSI that DDA distributes uniformly in the lungs and is detectable for at least 2 weeks post-i.pulmon. administration of CAF01. Both DDA and TDB co-localize with the endogenous phospholipids PC (34:1) in the positive ion mode and PS (38:4) in the negative ion mode. The phospholipid lysoPC (16:0) (36) and the sphingolipid ceramide (37) are well-known biomarkers of inflammation and local tissue damage, respectively. The absence of enhanced ceramide expression in the lungs of mice immunized with CAF01, and the lack of difference in the expression levels of LysoPC between the immunized mice and the negative control animal, suggest that i.pulmon. administration of CAF01 may be safe and does not induce any apparent inflammation in the lungs during the investigated time period. Using intranasal (66, 67) and i.pulmon. immunization (24, 68), our previous results have consistently shown the safety of immunization with the CAF01 adjuvant in preclinical animal models and most recently in phase I clinical trials in humans following intranasal immunization with a *Chlamydia* antigen (69). Therefore, in this study we did not expect any difference in the number of inflammatory cells among the immunization strategies and did not compare histopathological changes in lung tissue sections. BMP (22:6/22:6) is a negatively charged glycerophospholipid, which is primarily localized in the late endosomes/lysosomes (70). The BMP content is enriched in alveolar macrophages, as compared to other cell types, and it is primarily localized in phagosomes (35). BMP (22:6/22:6) has been shown to co-localize with the macrophage biomarker CD11b in ischemic brain tissue, and it is a reported biomarker for phagocytozing macrophages (34). The increased level of BMP after i.pulmon. administration of CAF01 liposomes measured in our study suggests an increased phagocytic activity in alveolar macrophages. In addition, the flow cytometry data demonstrates an increased number of alveolar macrophages 24 and 72 h after i.pulmon. administration of the H56/CAF01 vaccine. Hence, these results collectively suggest that after i.pulmon. administration of CAF01, there is (i) an increased alveolar macrophage activity, as well as (ii) an increased number of alveolar macrophages that takes up the vaccine. In a previous study, cholesteryl esters were shown to co-localize with BMP during resolution of cerebral inflammation due to phagocytosis of cholesterol-containing dead cells or cell debris (34). However, in our study, the BMP expression was not accompanied by increased levels of cholesteryl esters. The lack of cholesteryl ester expression in our study suggests that (i) there is no increased phagocytosis of dead cells following CAF01 administration in the lungs, and (ii) applying this administration route does not cause any measurable local inflammation.

In this study, we chose to investigate multiple time points by MSI post-i.pulmon. administration of CAF01, rather than including more animals in each group and fewer time points. We believe that this imaging method is a very robust method for evaluating even low doses/expression levels of proteins, lipids and their metabolites. However, to improve the statistical strength of our data, more biological replicates should be included in future studies. In addition, commonly investigated biomarkers for systemic inflammation, e.g., plasma C-reactive protein and interferon-γ inducible protein-10 concentrations, should be evaluated to investigate further the safety of pulmonary vaccination.

## Conclusion

In conclusion, we show that there are pronounced differences in the vaccine uptake by innate myeloid APCs in the lungs and the spleen and epithelial cells in the lungs, and in the activation state of APCs in the lung-draining lymph nodes after i.m. prime and i.pulmon. mucosal pull immunization with the H56/CAF01 vaccine, as compared to i.m. or i.pulmon. priming alone, which suggests activation of both innate and memory response by prime – pull immunization. Using phospholipid analysis by MALDI-MSI, we further conclude that airway mucosal immunization with H56/CAF01 is a safe immunization approach, which is critical to consider in the rational design of vaccines for pulmonary delivery. Overall, the differences in vaccine uptake by innate myeloid cells and activation of APCs among the different immunization strategies described here can be valuable to tailor vaccine-induced immunity.

## Data Availability Statement

The datasets generated for this study are available on request to the corresponding author.

## Ethics Statement

The animal study was reviewed and approved by the Danish National Experiment Inspectorate under permit 2016-15-0201-01026.

## Author Contributions

AT, DC, and CF designed the study. AT and FP performed the laboratory work and analyzed the data. AT, HH, DC, CJ, and CF interpreted the data. AT, FP, and CF drafted the manuscript. AT, HH, PA, DC, CJ, and CF provided scientific input throughout the study period and draft of the manuscript.

## Conflict of Interest

PA and DC are employed by Statens Serum Institut, a nonprofit government research facility, of which the CAF adjuvants and H56 are proprietary products. The remaining authors declare that the research was conducted in the absence of any commercial or financial relationships that could be construed as a potential conflict of interest.

## References

[1] WHO *Global Tuberculosis Report 2018.* Geneva: World Health Organization. (2018).

[2] Uplekar M Weil D Lonnroth K Jaramillo E Lienhardt C Dias HM WHO’s new end TB strategy. *Lancet.* (2015) 385:1799–801. 10.1016/S0140-6736(15)60570-0 25814376

[3] Foged C. Subunit vaccines of the future: the need for safe, customized and optimized particulate delivery systems. *Ther Deliv.* (2011) 2:1057–77. 10.4155/tde.11.68 22826868

[4] Delany I Rappuoli R De Gregorio E. Vaccines for the 21st century. *EMBO Mol Med.* (2014) 6:708–20. 10.1002/emmm.201403876 24803000 PMC4203350

[5] Beverley PC Sridhar S Lalvani A Tchilian EZ. Harnessing local and systemic immunity for vaccines against tuberculosis. *Mucosal Immunol.* (2014) 7:20–6. 10.1038/mi.2013.99 24253104

[6] Iqbal AJ Fisher EA Greaves DR. Inflammation-a critical appreciation of the role of myeloid cells. *Microbiol Spectr.* (2016) 4:MCHD-0027-2016.. 10.1128/microbiolspec.MCHD-0027-2016 27780018 PMC5119645

[7] Jaillon S Galdiero MR Del Prete D Cassatella MA Garlanda C Mantovani A. Neutrophils in innate and adaptive immunity. *Semin Immunopathol.* (2013) 35:377–94. 10.1007/s00281-013-0374-8 23553214

[8] Hashimoto D Miller J Merad M. Dendritic cell and macrophage heterogeneity in vivo. *Immunity.* (2011) 35:323–35. 10.1016/j.immuni.2011.09.007 21943488 PMC4520532

[9] Gerner MY Casey KA Kastenmuller W Germain RN. Dendritic cell and antigen dispersal landscapes regulate T cell immunity. *J Exp Med.* (2017) 214:3105–22. 10.1084/jem.20170335 28847868 PMC5626399

[10] Desch AN Henson PM Jakubzick CV. Pulmonary dendritic cell development and antigen acquisition. *Immunol Res.* (2013) 55:178–86. 10.1007/s12026-012-8359-6 22968708 PMC4153344

[11] Condon TV Sawyer RT Fenton MJ Riches DW. Lung dendritic cells at the innate-adaptive immune interface. *J Leukoc Biol.* (2011) 90:883–95. 10.1189/jlb.0311134 21807741 PMC3206474

[12] Blank F Stumbles PA Seydoux E Holt PG Fink A Rothen-Rutishauser B Size-dependent uptake of particles by pulmonary antigen-presenting cell populations and trafficking to regional lymph nodes. *Am J Respir Cell Mol Biol.* (2013) 49:67–77. 10.1165/rcmb.2012-0387OC 23492193

[13] Fromen CA Robbins GR Shen TW Kai MP Ting JP DeSimone JM. Controlled analysis of nanoparticle charge on mucosal and systemic antibody responses following pulmonary immunization. *Proc Natl Acad Sci USA.* (2015) 112:488–93. 10.1073/pnas.1422923112 25548169 PMC4299250

[14] Blank F Fytianos K Seydoux E Rodriguez-Lorenzo L Petri-Fink A von Garnier C Interaction of biomedical nanoparticles with the pulmonary immune system. *J Nanobiotechnology.* (2017) 15:6. 10.1186/s12951-016-0242-5 28069025 PMC5223535

[15] Aagaard C Hoang T Dietrich J Cardona PJ Izzo A Dolganov G A multistage tuberculosis vaccine that confers efficient protection before and after exposure. *Nat Med.* (2011) 17:189–94. 10.1038/nm.2285 21258338

[16] Knudsen NP Olsen A Buonsanti C Follmann F Zhang Y Coler RN Different human vaccine adjuvants promote distinct antigen-independent immunological signatures tailored to different pathogens. *Sci Rep.* (2016) 6:19570. 10.1038/srep19570 26791076 PMC4726129

[17] Lin PL Dietrich J Tan E Abalos RM Burgos J Bigbee C The multistage vaccine H56 boosts the effects of BCG to protect cynomolgus macaques against active tuberculosis and reactivation of latent *Mycobacterium tuberculosis* infection. *J Clin Invest.* (2012) 122:303–14. 10.1172/JCI46252 22133873 PMC3248283

[18] Davidsen J Rosenkrands I Christensen D Vangala A Kirby D Perrie Y Characterization of cationic liposomes based on dimethyldioctadecylammonium and synthetic cord factor from *M. tuberculosis* (trehalose 6,6’-dibehenate)-a novel adjuvant inducing both strong CMI and antibody responses. *Biochim Biophys Acta.* (2005) 1718:22–31. 10.1016/j.bbamem.2005.10.011 16321607

[19] Kamath AT Rochat AF Christensen D Agger EM Andersen P Lambert PH A liposome-based mycobacterial vaccine induces potent adult and neonatal multifunctional T cells through the exquisite targeting of dendritic cells. *PLoS One.* (2009) 4:e5771. 10.1371/journal.pone.0005771 19492047 PMC2685976

[20] van Dissel JT Joosten SA Hoff ST Soonawala D Prins C Hokey DA A novel liposomal adjuvant system, CAF01, promotes long-lived *Mycobacterium tuberculosis*-specific T-cell responses in human. *Vaccine.* (2014) 32:7098–107. 10.1016/j.vaccine.2014.10.036 25454872

[21] Roman VR Jensen KJ Jensen SS Leo-Hansen C Jespersen S da Silva Te D Therapeutic vaccination using cationic liposome-adjuvanted HIV type 1 peptides representing HLA-supertype-restricted subdominant T cell epitopes: safety, immunogenicity, and feasibility in Guinea-Bissau. *AIDS Res Hum Retroviruses.* (2013) 29:1504–12. 10.1089/AID.2013.0076 23634822

[22] Karlsson I Brandt L Vinner L Kromann I Andreasen LV Andersen P Adjuvanted HLA-supertype restricted subdominant peptides induce new T-cell immunity during untreated HIV-1-infection. *Clin Immunol.* (2013) 146:120–30. 10.1016/j.clim.2012.12.005 23314272

[23] Woodworth JS Christensen D Cassidy JP Agger EM Mortensen R Andersen P. Mucosal boosting of H56:CAF01 immunization promotes lung-localized T cells and an accelerated pulmonary response to *Mycobacterium tuberculosis* infection without enhancing vaccine protection. *Mucosal Immunol.* (2019) 12:816–26. 10.1038/s41385-019-0145-5 30760832

[24] Thakur A Rodriguez-Rodriguez C Saatchi K Rose F Esposito T Nosrati Z Dual-Isotope SPECT/CT imaging of the tuberculosis subunit vaccine H56/CAF01: induction of strong systemic and mucosal IgA and T-cell responses in mice upon subcutaneous prime and intrapulmonary boost immunization. *Front Immunol.* (2018) 9:2825. 10.3389/fimmu.2018.02825 30555488 PMC6284049

[25] Woodland DL. Jump-starting the immune system: prime-boosting comes of age. *Trends Immunol.* (2004) 25:98–104. 10.1016/j.it.2003.11.009 15102369

[26] Singer BD Mock JR D’Alessio FR Aggarwal NR Mandke P Johnston L Flow-cytometric method for simultaneous analysis of mouse lung epithelial, endothelial, and hematopoietic lineage cells. *Am J Physiol Lung Cell Mol Physiol.* (2016) 310:L796–801. 10.1152/ajplung.00334.2015 26944088 PMC4867353

[27] Misharin AV Morales-Nebreda L Mutlu GM Budinger GR Perlman H. Flow cytometric analysis of macrophages and dendritic cell subsets in the mouse lung. *Am J Respir Cell Mol Biol.* (2013) 49:503–10. 10.1165/rcmb.2013-0086MA 23672262 PMC3824047

[28] Pulendran B. The varieties of immunological experience: of pathogens, stress, and dendritic cells. *Annu Rev Immunol.* (2015) 33:563–606. 10.1146/annurev-immunol-020711-075049 25665078

[29] Bouschen W Schulz O Eikel D Spengler B. Matrix vapor deposition/recrystallization and dedicated spray preparation for high-resolution scanning microprobe matrix-assisted laser desorption/ionization imaging mass spectrometry (SMALDI-MS) of tissue and single cells. *Rapid Commun Mass Spectrom.* (2010) 24:355–64. 10.1002/rcm.4401 20049881

[30] Janfelt C Wellner N Leger PL Kokesch-Himmelreich J Hansen SH Charriaut-Marlangue C Visualization by mass spectrometry of 2-dimensional changes in rat brain lipids, including N-acylphosphatidylethanolamines, during neonatal brain ischemia. *FASEB J.* (2012) 26:2667–73. 10.1096/fj.11-201152 22389441

[31] Schramm T Hester A Klinkert I Both JP Heeren RM Brunelle A imzML–a common data format for the flexible exchange and processing of mass spectrometry imaging data. *J Proteomics.* (2012) 75:5106–10. 10.1016/j.jprot.2012.07.026 22842151

[32] Robichaud G Garrard KP Barry JA Muddiman DC. MSiReader: an open-source interface to view and analyze high resolving power MS imaging files on Matlab platform. *J Am Soc Mass Spectrom.* (2013) 24:718–21. 10.1007/s13361-013-0607-z 23536269 PMC3693088

[33] Schmidt ST Khadke S Korsholm KS Perrie Y Rades T Andersen P The administration route is decisive for the ability of the vaccine adjuvant CAF09 to induce antigen-specific CD8(+) T-cell responses: the immunological consequences of the biodistribution profile. *J Control Release.* (2016) 239:107–17. 10.1016/j.jconrel.2016.08.034 27574990 PMC5041310

[34] Nielsen MM Lambertsen KL Clausen BH Meyer M Bhandari DR Larsen ST Mass spectrometry imaging of biomarker lipids for phagocytosis and signalling during focal cerebral ischaemia. *Sci Rep.* (2016) 6:39571. 10.1038/srep39571 28004822 PMC5177920

[35] Akgoc Z Iosim S Seyfried TN. Bis(monoacylglycero)phosphate as a Macrophage enriched phospholipid. *Lipids.* (2015) 50:907–12. 10.1007/s11745-015-4045-5 26205346

[36] Qin X Qiu C Zhao L. Lysophosphatidylcholine perpetuates macrophage polarization toward classically activated phenotype in inflammation. *Cell Immunol.* (2014) 289:185–90. 10.1016/j.cellimm.2014.04.010 24841857

[37] Kurz J Parnham MJ Geisslinger G Schiffmann S. Ceramides as novel disease biomarkers. *Trends Mol Med.* (2019) 25:20–32. 10.1016/j.molmed.2018.10.009 30477968

[38] von Halling Laier C Gibson B Moreno JAS Rades T Hook S Nielsen LH Microcontainers for protection of oral vaccines, in vitro and in vivo evaluation. *J Control Release.* (2018) 294:91–101. 10.1016/j.jconrel.2018.11.030 30550938

[39] Blank F Stumbles P von Garnier C. Opportunities and challenges of the pulmonary route for vaccination. *Expert Opin Drug Deliv.* (2011) 8:547–63. 10.1517/17425247.2011.565326 21438741

[40] Khader SA Bell GK Pearl JE Fountain JJ Rangel-Moreno J Cilley GE IL-23 and IL-17 in the establishment of protective pulmonary CD4+ T cell responses after vaccination and during *Mycobacterium tuberculosis* challenge. *Nat Immunol.* (2007) 8:369–77. 10.1038/ni1449 17351619

[41] Srivastava S Ernst JD. Cutting edge: direct recognition of infected cells by CD4 T cells is required for control of intracellular *Mycobacterium tuberculosis* in vivo. *J Immunol.* (2013) 191:1016–20. 10.4049/jimmunol.1301236 23817429 PMC3725655

[42] Jeyanathan M Shao Z Yu X Harkness R Jiang R Li J AdHu5Ag85A respiratory mucosal boost immunization enhances protection against pulmonary tuberculosis in BCG-primed non-human primates. *PLoS One.* (2015) 10:e0135009. 10.1371/journal.pone.0135009 26252520 PMC4529167

[43] Satti I Meyer J Harris SA Manjaly Thomas ZR Griffiths K Antrobus RD Safety and immunogenicity of a candidate tuberculosis vaccine MVA85A delivered by aerosol in BCG-vaccinated healthy adults: a phase 1, double-blind, randomised controlled trial. *Lancet Infect Dis.* (2014) 14:939–46. 10.1016/S1473-3099(14)70845-X 25151225 PMC4178237

[44] Perdomo C Zedler U Kuhl AA Lozza L Saikali P Sander LE Mucosal BCG vaccination induces protective lung-resident memory T cell populations against tuberculosis. *MBio.* (2016) 7:e01686-16. 10.1128/mBio.01686-16 27879332 PMC5120139

[45] Foged C. Thermostable subunit vaccines for pulmonary delivery: how close are we? *Curr Pharm Des.* (2016) 22:2561–76. 10.2174/1381612822666160202141603 26831645

[46] Thakur A Andrea A Mikkelsen H Woodworth JS Andersen P Jungersen G Targeting the mincle and TLR3 receptor using the dual agonist cationic adjuvant formulation 9 (CAF09) induces humoral and polyfunctional memory T cell responses in calves. *PLoS One.* (2018) 13:e0201253. 10.1371/journal.pone.0201253 30063728 PMC6067743

[47] Mangtani P Abubakar I Ariti C Beynon R Pimpin L Fine PE Protection by BCG vaccine against tuberculosis: a systematic review of randomized controlled trials. *Clin Infect Dis.* (2014) 58:470–80. 10.1093/cid/cit790 24336911

[48] de Bree LCJ Koeken V Joosten LAB Aaby P Benn CS van Crevel R Non-specific effects of vaccines: current evidence and potential implications. *Semin Immunol.* (2018) 39:35–43. 10.1016/j.smim.2018.06.002 30007489

[49] Guilliams M Lambrecht BN Hammad H. Division of labor between lung dendritic cells and macrophages in the defense against pulmonary infections. *Mucosal Immunol.* (2013) 6:464–73. 10.1038/mi.2013.14 23549447

[50] Blom RAM Amacker M van Dijk RM Moser C Stumbles PA Blank F Pulmonary delivery of virosome-bound antigen enhances antigen-specific CD4(+) T cell proliferation compared to liposome-bound or soluble antigen. *Front Immunol.* (2017) 8:359. 10.3389/fimmu.2017.00359 28439267 PMC5383731

[51] Jakubzick C Tacke F Llodra J van Rooijen N Randolph GJ. Modulation of dendritic cell trafficking to and from the airways. *J Immunol.* (2006) 176:3578–84. 10.4049/jimmunol.176.6.3578 16517726

[52] Hufford MM Richardson G Zhou H Manicassamy B Garcia-Sastre A Enelow RI Influenza-infected neutrophils within the infected lungs act as antigen presenting cells for anti-viral CD8(+) T cells. *PLoS One.* (2012) 7:e46581. 10.1371/journal.pone.0046581 23056353 PMC3466305

[53] Blomgran R Ernst JD. Lung neutrophils facilitate activation of naive antigen-specific CD4+ T cells during *Mycobacterium tuberculosis* infection. *J Immunol.* (2011) 186:7110–9. 10.4049/jimmunol.1100001 21555529 PMC3376160

[54] Palgen JL Tchitchek N Elhmouzi-Younes J Delandre S Namet I Rosenbaum P Prime and boost vaccination elicit a distinct innate myeloid cell immune response. *Sci Rep.* (2018) 8:3087. 10.1038/s41598-018-21222-2 29449630 PMC5814452

[55] Wosen JE Mukhopadhyay D Macaubas C Mellins ED. Epithelial MHC Class II expression and its role in antigen presentation in the gastrointestinal and respiratory tracts. *Front Immunol.* (2018) 9:2144. 10.3389/fimmu.2018.02144 30319613 PMC6167424

[56] Hershberg RM Mayer LF. Antigen processing and presentation by intestinal epithelial cells - polarity and complexity. *Immunol Today.* (2000) 21:123–8. 10.1016/s0167-5699(99)01575-3 10689299

[57] Kumari M Saxena RK. Relative efficacy of uptake and presentation of *Mycobacterium bovis* BCG antigens by type I mouse lung epithelial cells and peritoneal macrophages. *Infect Immun.* (2011) 79:3159–67. 10.1128/IAI.05406-11 21646448 PMC3147596

[58] Corbiere V Dirix V Norrenberg S Cappello M Remmelink M Mascart F. Phenotypic characteristics of human type II alveolar epithelial cells suitable for antigen presentation to T lymphocytes. *Respir Res.* (2011) 12:15. 10.1186/1465-9921-12-15 21261956 PMC3033824

[59] Rothermel AL Wang Y Schechner J Mook-Kanamori B Aird WC Pober JS Endothelial cells present antigens in vivo. *BMC Immunol.* (2004) 5:5. 10.1186/1471-2172-5-5 15113397 PMC394319

[60] Perez VL Henault L Lichtman AH. Endothelial antigen presentation: stimulation of previously activated but not naive TCR-transgenic mouse T cells. *Cell Immunol.* (1998) 189:31–40. 10.1006/cimm.1998.1362 9758692

[61] Sanchez-Abarca LI Alvarez-Laderas I Diez Campelo M Caballero-Velazquez T Herrero C Muntion S Uptake and delivery of antigens by mesenchymal stromal cells. *Cytotherapy.* (2013) 15:673–8. 10.1016/j.jcyt.2013.01.216 23522868

[62] Kamath AT Mastelic B Christensen D Rochat AF Agger EM Pinschewer DD Synchronization of dendritic cell activation and antigen exposure is required for the induction of Th1/Th17 responses. *J Immunol.* (2012) 188:4828–37. 10.4049/jimmunol.1103183 22504654

[63] Thakur A Ingvarsson PT Schmidt ST Rose F Andersen P Christensen D Immunological and physical evaluation of the multistage tuberculosis subunit vaccine candidate H56/CAF01 formulated as a spray-dried powder. *Vaccine.* (2018) 36:3331–9. 10.1016/j.vaccine.2018.04.055 29699790

[64] Ferguson CN Fowler JW Waxer JF Gatti RA Loo JA. Mass spectrometry-based tissue imaging of small molecules. *Adv Exp Med Biol.* (2014) 806:283–99. 10.1007/978-3-319-06068-2_12 24952187 PMC4183127

[65] Berry KA Li B Reynolds SD Barkley RM Gijon MA Hankin JA MALDI imaging MS of phospholipids in the mouse lung. *J Lipid Res.* (2011) 52:1551–60. 10.1194/jlr.M015750 21508254 PMC3137021

[66] Ciabattini A Prota G Christensen D Andersen P Pozzi G Medaglini D. Characterization of the antigen-specific CD4(+) T cell response induced by prime-boost strategies with CAF01 and CpG adjuvants administered by the intranasal and subcutaneous routes. *Front Immunol.* (2015) 6:430. 10.3389/fimmu.2015.00430 26379666 PMC4551867

[67] Lorenzen E Follmann F Boje S Erneholm K Olsen AW Agerholm JS Intramuscular priming and intranasal boosting induce strong genital immunity through secretory IgA in minipigs infected with chlamydia trachomatis. *Front Immunol.* (2015) 6:628. 10.3389/fimmu.2015.00628 26734002 PMC4679855

[68] Thakur A Rose F Ansari SR Koch P Martini V Ovesen SL Design of gadoteridol-loaded cationic liposomal adjuvant CAF01 for MRI of lung deposition of intrapulmonary administered particles. *Mol Pharm.* (2019) 16:4725–37. 10.1021/acs.molpharmaceut.9b00908 31539263

[69] Abraham S Juel HB Bang P Cheeseman HM Dohn RB Cole T Safety and immunogenicity of the chlamydia vaccine candidate CTH522 adjuvanted with CAF01 liposomes or aluminium hydroxide: a first-in-human, randomised, double-blind, placebo-controlled, phase 1 trial. *Lancet Infect Dis.* (2019) 19:1091–100. 10.1016/S1473-3099(19)30279-8 31416692

[70] van der Goot FG Gruenberg J. Intra-endosomal membrane traffic. *Trends Cell Biol.* (2006) 16:514–21. 10.1016/j.tcb.2006.08.003 16949287

