# Supplementary material for: Intrapulmonary (i.pulmon.) Pull Immunization With the Tuberculosis Subunit Vaccine Candidate H56/CAF01 After Intramuscular (i.m.) Priming Elicits a Distinct Innate Myeloid Response and Activation of Antigen-Presenting Cells Than i.m. or i.pulmon. Prime Immunization Alone
Source: Front Immunol. 2020 May 7;11:803. doi: 10.3389/fimmu.2020.00803 (PMC7221191; doi:10.3389/fimmu.2020.00803)
Supplement: Supplementary file 1 [file Data_Sheet_1.docx]

**Supplementary information**

as part of the manuscript

**Intrapulmonary (i.pulmon.) pull immunization with the tuberculosis subunit vaccine candidate H56/CAF01 after intramuscular (i.m.) priming elicits a distinct innate myeloid response and activation of antigen-presenting cells than i.m. or i.pulmon. prime immunization alone**

**Aneesh Thakur^1*^, Fernanda Endringer Pinto^2^, Harald Severin Hansen^3^, Peter Andersen^4^, Dennis Christensen^4^, Christian Janfelt^1^, and Camilla Foged^1^**

*^1^ Department of Pharmacy, Faculty of Health and Medical Sciences, University of Copenhagen, Universitetsparken 2, 2100 Copenhagen Ø, Denmark*

*^2^* *Department of Chemistry, Federal University of Espírito Santo, 29075-910, Vitória, Brazil*

*^3^* *Department of Drug Design and Pharmacology, Faculty of Health and Medical Sciences, University of Copenhagen, Universitetsparken 2, 2100 Copenhagen Ø, Denmark*

*^4^ Department of Infectious Disease Immunology, Statens Serum Institut, Artillerivej 5, 2300 Copenhagen S, Denmark*

*Corresponding author: Aneesh Thakur, Department of Pharmacy, Faculty of Health and Medical Sciences, University of Copenhagen, Universitetsparken 2, DK-2100 Copenhagen Ø, Denmark, Phone/Fax: + 45 35 33 39 38/+45 35 33 60 01, Email: aneesh.thakur@sund.ku.dk

**Index**

**Page**

1. Table S1. Flow cytometry panels, antibodies, and staining reagents.
2. Table S2: Average number of lung and spleen cells per group at different time points.
3. Figure S1. Gating strategy applied to identify different immune cells in the lungs by flow cytometry.
4. Figure S2. Gating strategy applied to identify epithelial, endothelial, hematopoietic lineage, and lineage-negative cells in the lungs by flow cytometry.
5. Figure S3. Gating strategy applied to identify different immune cells in the spleen or the lymph nodes by flow cytometry.
6. Figure S4. Distribution of phosphatidylcholine [PC (34:1)] in mice lungs following intrapulmonary administration of CAF01.
7. Figure S5. Distribution and signal intensity of trehalose 6,6'-dibehenate (TDB) in mice lungs following intrapulmonary administration of CAF01.
8. Figure S6. Distribution of phosphatidylserine [PS (38:4)] in mice lungs after intrapulmonary administration of CAF01.
9. Figure S7. Distribution and signal intensity of lysophosphatidylcholine (LysoPC (16:0) in mice lungs following intrapulmonary administration of CAF01.

Table S1: Flow cytometry panels, antibodies, and staining reagents.

All antibodies for flow cytometry were obtained from BD (BD Biosciences, Lyngby, Denmark).

| **Antibody** | **Clone** | **Isotype** | **Conjugate** |
| --- | --- | --- | --- |
| **Panel 1 (Lungs)** | | | |
| CD11b | M1770 | IgG2b κ | BB515 |
| CD11c | HL3 | IgG1 λ2 | BUV737 |
| CD19 | 1D3 | IgG2a κ | BB700 |
| CD64 | X54-5./7.1 | IgG1 κ | BV786 |
| CD86 | GL1 | IgG2a κ | BV480 |
| CD103 | M290 | IgG2a κ | PE-CF594 |
| F4/80 | T45-2342 | IgG2a κ | PE |
| Ly6G | 1A8 | IgG2a κ | PE-Cy7 |
| Ly6C | AL-21 | IgM κ | BV421 |
| Siglec F | E50-2440 | IgG2a κ | BV711 |
| I-A/I-E | 2G9 | IgG2a κ | BUV395 |
| **Panel 2 (Lungs)** | | | |
| CD31 | MEC 13.3 | IgG2a κ | BUV737 |
| CD45 | 30-F11 | IgG2b κ | BB515 |
| CD74 | In-1 | IgG2b κ | BV605 |
| CD326 | G8.8 | IgG2a κ | BV421 |
| Podoplanin | PE8.1.1 | IgG2 | PE |
| I-A/I-E | 2G9 | IgG2a κ | BUV395 |
| **Panel 3 (Spleen and lymph nodes)** | | | |
| CD8α | 53-6.7 | IgG2a κ | PE-CF594 |
| CD11b | M1770 | IgG2b κ | BB515 |
| CD11c | HL3 | IgG1 λ2 | BUV737 |
| CD19 | 1D3 | IgG2a κ | BV605 |
| CD64 | X54-5./7.1 | IgG1 κ | BV786 |
| CD86 | GL1 | IgG2a κ | BV480 |
| F4/80 | T45-2342 | IgG2a κ | PE |
| Ly6G | 1A8 | IgG2a κ | PE-Cy7 |
| Ly6C | AL-21 | IgM κ | BV421 |
| Siglec F | E50-2440 | IgG2a κ | BV711 |
| I-A/I-E | 2G9 | IgG2a κ | BUV395 |

Table S2: Average number of lung and spleen cells per group at different time points.

|  | **3h** | **24h** | **72h** |
| --- | --- | --- | --- |
| **Lungs** |  |  |  |
| **i.m.** |  |  |  |
| H56 | 475800 | 264300 | 316800 |
| H56/CAF01 | 338100 | 255150 | 300900 |
| **i.pulmon.** |  |  |  |
| H56 | 210600^*^ | 209040 | 235200 |
| H56/CAF01 | 291060 | 270180 | 809400^****^ |
| **i.m./i.pulmon.** |  |  |  |
| H56 | 278280 | 350400 | 454800 |
| H56/CAF01 | 291300 | 402600 | 540960^*¤^ |
| **Spleen** |  |  |  |
| **i.m.** |  |  |  |
| H56 | 35808 | 169200 | 166200 |
| H56/CAF01 | 37869 | 349200 | 379890 |
| **i.pulmon.** |  |  |  |
| H56 | 187800 | 136800 | 177000 |
| H56/CAF01 | 189600 | 440700 | 383700 |
| **i.m./i.pulmon.** |  |  |  |
| H56 | 192600 | 243000 | 642600^*^ |
| H56/CAF01 | 237600 | 262200 | 399600 |

Data points represent n = 2 (for H56) and n = 4 (for H56 + CAF01). **p* < 0.05 and *****p* < 0.0001 vs. i.m. immunization and ^¤^*p* < 0.05 vs. i.pulmon. immunization *via* two-way ANOVA with Tukey’s post-test.

**
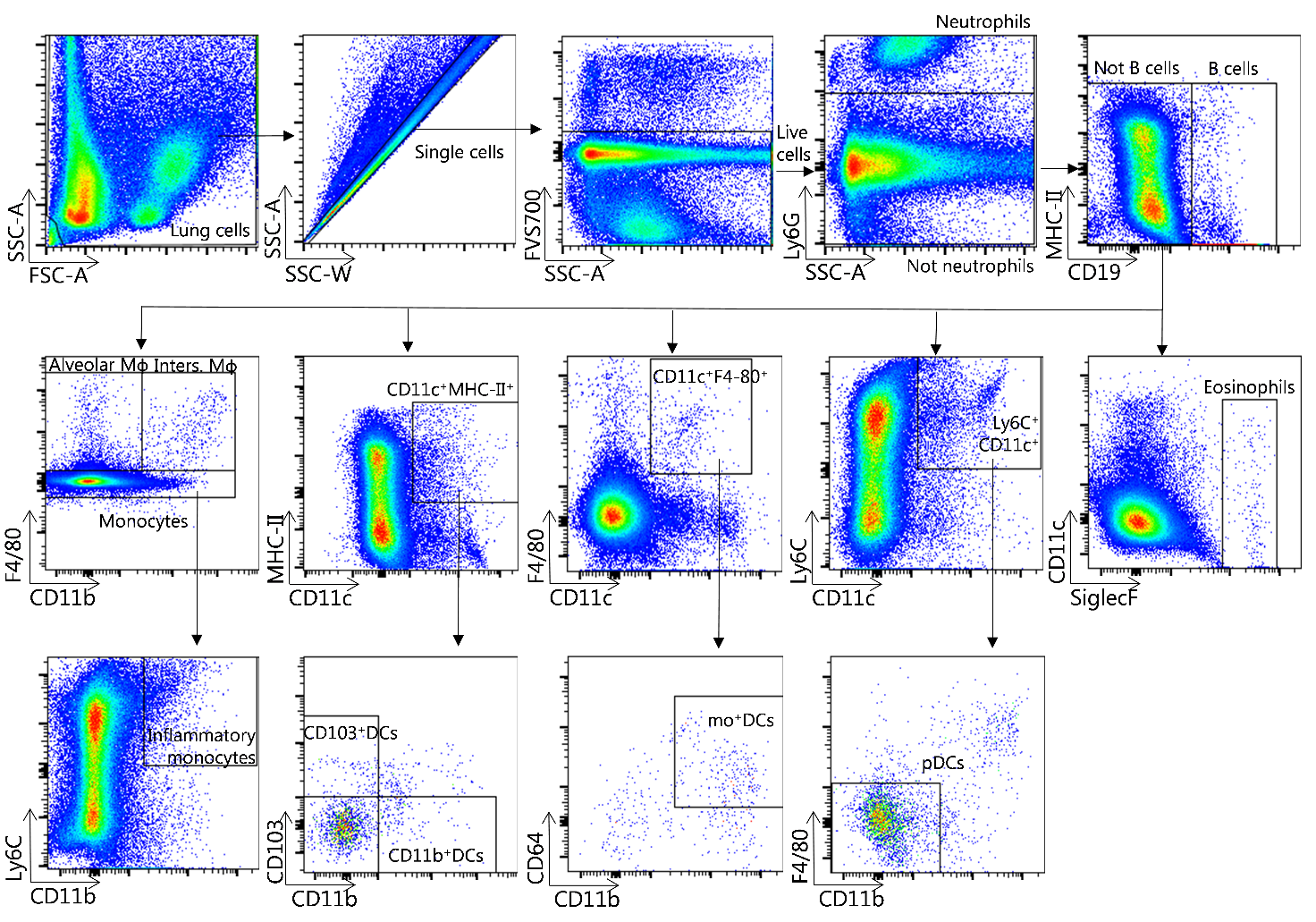
**

Figure S1. Gating strategy applied to identify different immune cell subsets in the lungs by flow cytometry. Cells of interest were gated according to their forward (FSC) and sideward (SSC) scatter, followed by single cell gating. From the live cell population, neutrophils were identified according to their Ly6G expression. B cells were identified from the non-neutrophils as the CD19^+^ population. From the non-B cells (CD19^-^ population), alveolar macrophages (F4/80^+^CD11b^-^), interstitial macrophages (F4/80^+^CD11b^+^), moDCs (CD11c^+^F4/80^+^CD11b^+^CD64^+^), CD11b^+^ DCs (CD11c^+^CD11b^+^), CD103^+^ DCs (CD11c^+^CD11b^-^CD103^+^), pDCs (CD11c^+^Ly6C^+^F4/80^-^CD11b^-^), eosinophils (SiglecF^+^), and inflammatory monocytes (Ly6C^+^CD11b^+^) were identified. The H56^+^ and vaccine^+^ (H56^+^/CAF01^+^) cells were identified among these cellular subsets based on the Alexa Fluor® 647 (H56) and DiR (CAF01) signals.

**
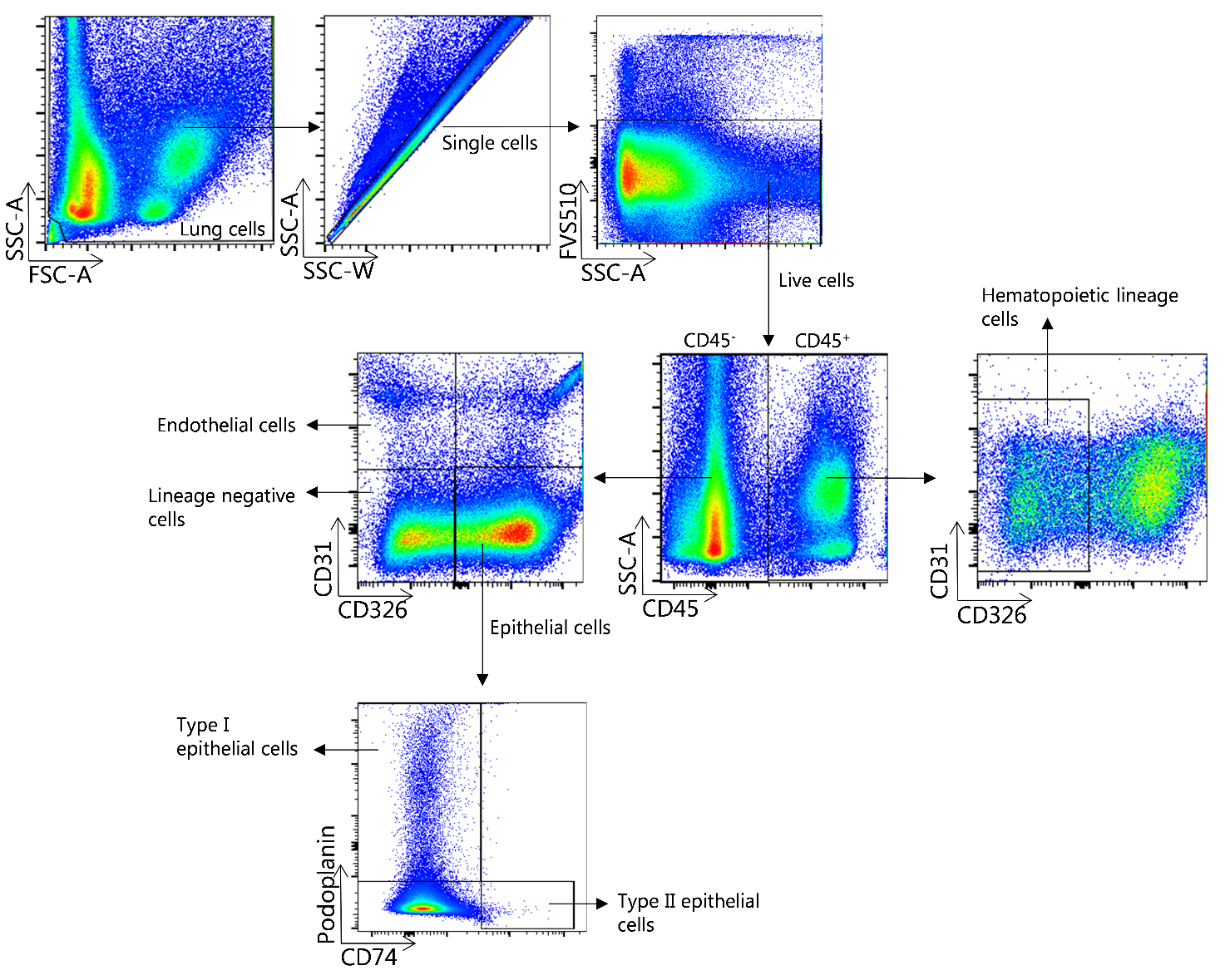
**

Figure S2. Gating strategy applied to identify epithelial, endothelial, hematopoietic lineage, and lineage-negative cell subsets in the lungs by flow cytometry. Cells of interest were gated according to their forward (FSC) and sideward (SSC) scatter signal followed by single cell gating. From the live cell populations, the CD45 status of the cells was first assessed, and the CD326 and CD31 statuses were subsequently assessed within the CD45^-^ and CD45^+^ populations to identify epithelial cells (CD45^-^CD31^-^CD326^+^), endothelial cells (CD45^-^CD31^+^CD326^-^), hematopoietic lineage cells (CD45^+^CD31^-^CD326^-^), and lineage negative cells (CD45^-^CD31^-^CD326^-^) in the lungs. Within the epithelial cell population, type I (CD45^-^CD31^-^CD326^+^CD74^-^Podoplanin^+^) and type II epithelial cells (CD45^-^CD31^-^CD326^+^CD74^+^Podoplanin^-^) were identified. H56^+^ and vaccine^+^ (H56^+^/CAF01^+^) cells were identified among these cellular subsets based on the Alexa Fluor® 647 (H56) and DiR (CAF01) signals.


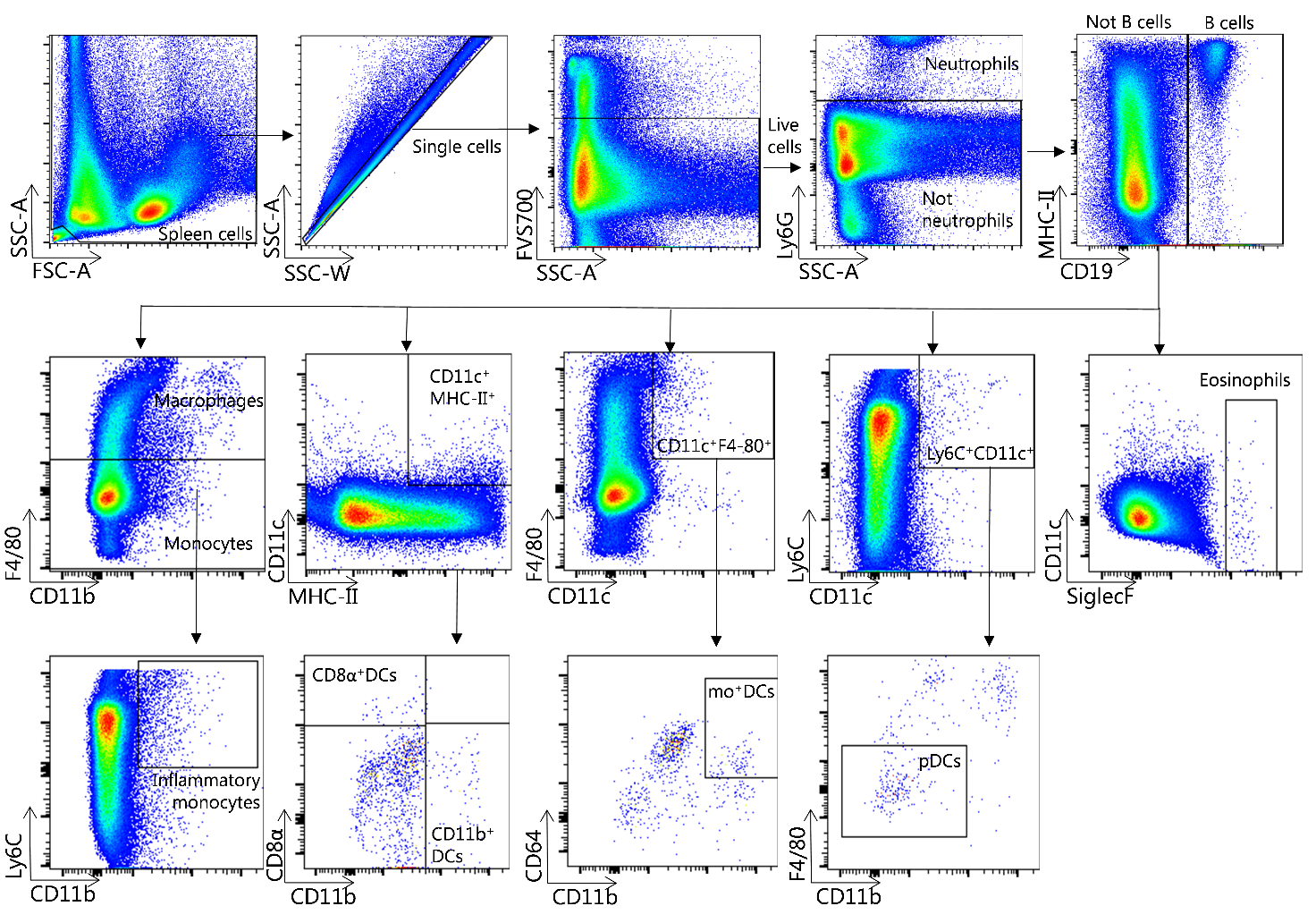


Figure S3. Gating strategy applied to identify different immune cell subsets in the spleen or the lymph nodes by using flow cytometry. Cells of interest were gated according to forward (FSC) and sideward (SSC) scatter followed by single cell gating. From the live cell population, neutrophils were identified according to their expression pf Ly6G. B cells were identified from the non-neutrophils as the CD19^+^ population. From the non-B cells (CD19^-^ population), moDCs (CD11c^+^F4/80^+^CD11b^+^CD64^+^), CD8α^+^ DCs (CD11c^+^CD11b^-^CD8α ^+^), CD11b^+^ DCs (CD11c^+^CD11b^+^), pDCs (CD11c^+^Ly6C^+^F4/80^-^CD11b^-^), macrophages (F4/80^+^CD11b^+^), eosinophils (SiglecF^+^), and inflammatory monocytes (Ly6C^+^CD11b^+^) were identified. The H56^+^ and vaccine^+^ (H56^+^/CAF01^+^) cells were identified among these cellular subsets based on the Alexa Fluor® 647 (H56) and DiR (CAF01) signals.


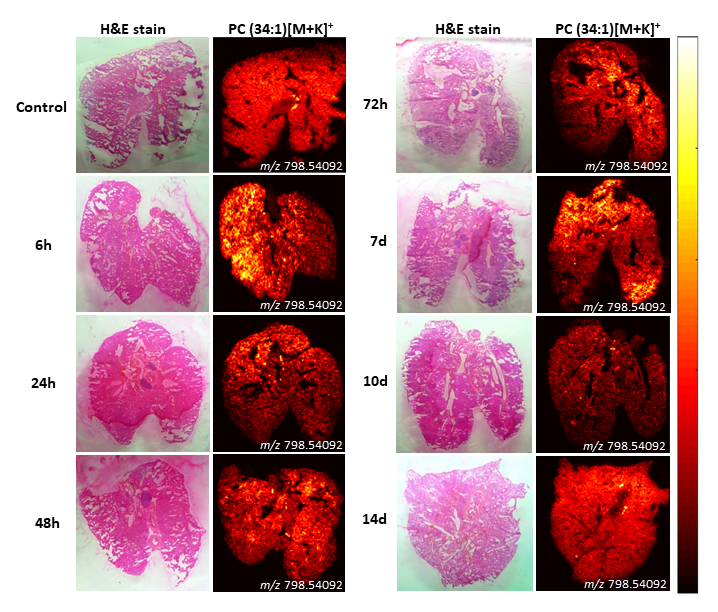


Figure S4. Distribution of phosphatidylcholine [PC (34:1)] in cryo-sections of mouse lungs following intrapulmonary administration of CAF01. Hematoxylin and eosin (H&E) stainings (left panels) and matrix-assisted laser desorption/ionization mass spectrometry (MALDI-MS) imaging-based distribution (right panels) of PC (34:1) [M+K]^+^ (m/z 798.54092) in cryo-sections of mice lungs at 6 h, 24 h, 48 h, 72 h, 7 d, 10 d, and 14 d following intrapulmonary immunization with CAF01. Untreated mice served as negative control. All images were measured in the positive ion mode by MALDI-MS imaging at a pixel size of 100 μm.


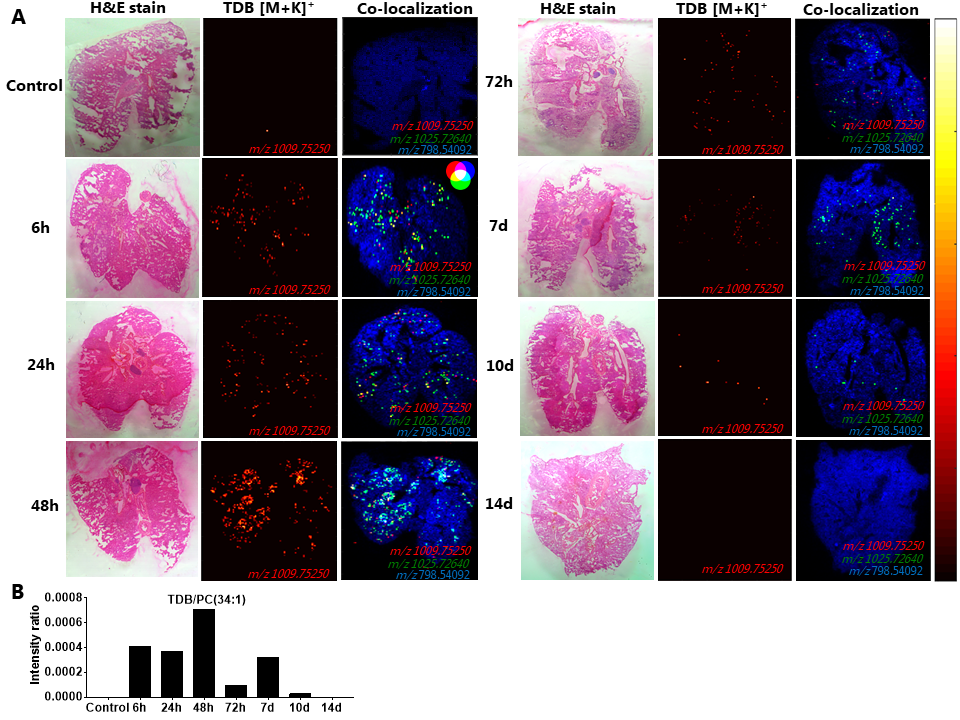


Figure S5. Distribution and signal intensity of trehalose 6,6'-dibehenate (TDB) in mouse lungs following intrapulmonary (i.pulmon.) administration of CAF01. BALB/c mice were immunized once with CAF01 *via* the i.pulmon. route, and matrix-assisted laser desorption/ionization mass spectrometry imaging (MALDI-MSI) was performed on lung cryo-sections at 6 h, 24 h, 48 h, 72 h, 7 d, 10 d, and 14 d after immunization. Untreated mice served as negative control. (A) Hematoxylin and eosin (H&E) staining (left panels), MALDI-MSI-based distribution of TDB [M+K]^+^ (m/z 1025.72640 ± 0.002, middle panels)), and mass spectrometry (MS) co-localization image (right panels) of TDB [M+Na]^+^ (m/z 1009.75250) (red), TDB [M+K]^+^ (m/z 1025.72640) (green), and PC(34:1) [M+K]^+^ (m/z 798.54092) (blue) in lung cryo-sections at different time points after i.pulmon. administration of CAF01. (B) Signal intensity ratios between TDB (m/z 1025.72640) and PC (34:1) at different time points of the study, which were calculated after drawing a region of interest (ROI) across the lung sections and comparing the MS signal intensities in the respective ROIs. All images were measured in the positive ion mode by using MALDI-MSI at a pixel size of 100 μm.


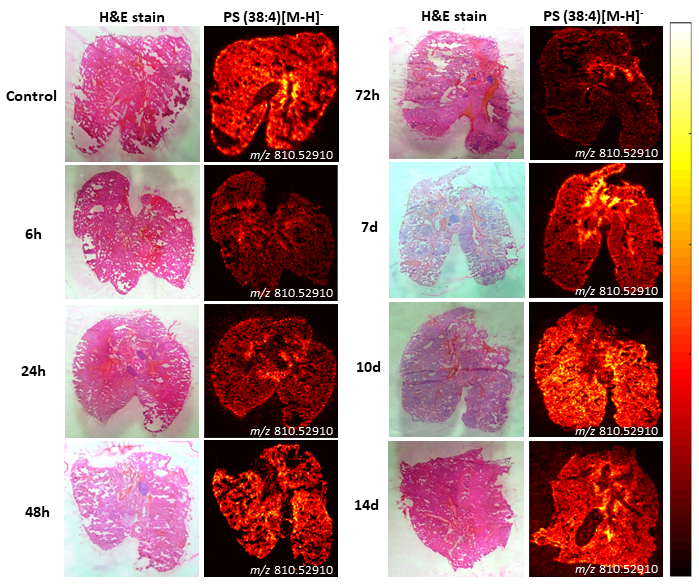


Figure S6. Distribution of phosphatidylserine [PS (38:4)] in mouse lungs following intrapulmonary (i.pulmon.) administration of CAF01. Hematoxylin and eosin (H&E) staining (left panels) and matrix-assisted laser desorption/ionization mass spectrometry (MALDI-MS) imaging-based distribution of PS (38:4) [M-H]^-^ (m/z 810.52910, right panels) in cryo-sections of mice lungs at 6 h, 24 h, 48 h, 72 h, 7 d, 10 d, and 14 d following i.pulmon. immunization with CAF01. Untreated mice served as negative control. All images were measured in the negative ion mode by MALDI-MS imaging at a pixel size of 100 μm.


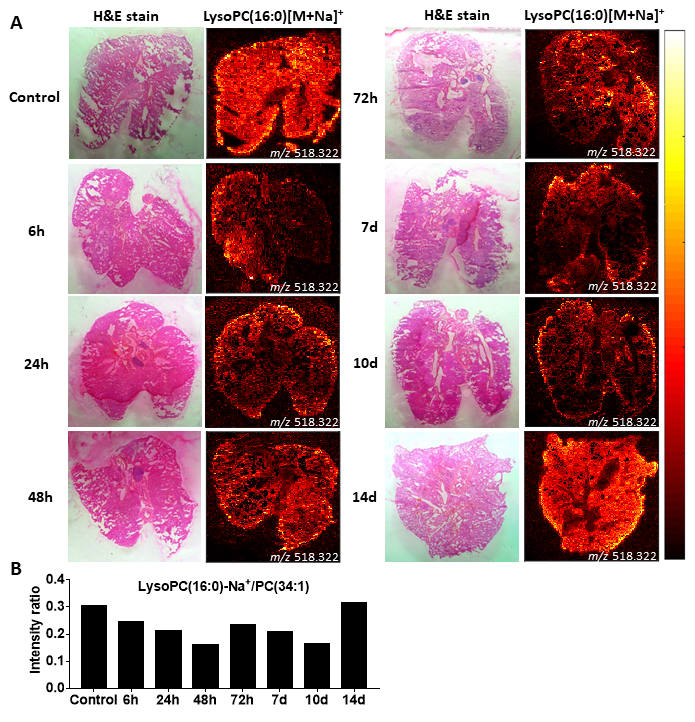


Figure S7. Distribution and signal intensity of lysophosphatidylcholine [LysoPC (16:0)] in mouse lungs following intrapulmonary (i.pulmon.) administration of CAF01. BALB/c mice were immunized once with CAF01 *via* the i.pulmon. route, and matrix-assisted laser desorption/ionization mass spectrometry imaging (MALDI-MSI) was performed on lung cryo-sections at 6 h, 24 h, 48 h, 72 h, 7 d, 10 d, and 14 d after immunization. Untreated mice served as negative control. (A) Hematoxylin and eosin (H&E) staining (left panels) and MALDI-MSI-based distribution (right panels) of LysoPC (16:0) (m/z 518.322 ± 0.002) in the lungs at different time points after i.pulmon. administration of CAF01. (B) Signal intensity ratio between LysoPC (m/z 518.322 ± 0.002) and PC (34:1) [M+K]^+^ (m/z 798.541) at different time points of the study, which were calculated after drawing a region of interest (ROI) across the lung sections and comparing the MS signal intensities in the respective ROIs. All images were measured in the positive ion mode by MALDI-MSI at a pixel size of 100 μm.
